# *Tagetes* spp. Essential Oils and Other Extracts: Chemical Characterization and Biological Activity

**DOI:** 10.3390/molecules23112847

**Published:** 2018-11-01

**Authors:** Bahare Salehi, Marco Valussi, Maria Flaviana Bezerra Morais-Braga, Joara Nalyda Pereira Carneiro, Antonio Linkoln Alves Borges Leal, Henrique Douglas Melo Coutinho, Sara Vitalini, Dorota Kręgiel, Hubert Antolak, Mehdi Sharifi-Rad, Nathália Cristina Cirone Silva, Zubaida Yousaf, Miquel Martorell, Marcello Iriti, Simone Carradori, Javad Sharifi-Rad

**Affiliations:** 1 Medical Ethics and Law Research Center, Shahid Beheshti University of Medical Sciences, Tehran 88777539, Iran; bahar.salehi007@gmail.com; 2 Student Research Committee, Shahid Beheshti University of Medical Sciences, Tehran 22439789, Iran; 3 European Herbal and Traditional Medicine Practitioners Association (EHTPA), 25 Lincoln Close, Tewkesbury GL20 5TY, UK; marco.officinalessinia@gmail.com; 4 Laboratory of Microbiology and Molecular Biology—LMBM, Regional University of Cariri—URCA, Crato, CE 63105-000, Brazil; flavianamoraisb@yahoo.com.br (M.F.B.M.-B.); nalyda_05@hotmail.com (J.N.P.C.); antoniolinkoln@hotmail.com (A.L.A.B.L.); hdmcoutinho@gmail.com (H.D.M.C.); 5 Department of Agricultural and Environmental Sciences, Milan State University, via G. Celoria 2, 20133 Milan, Italy; sara.vitalini@unimi.it (S.V.); marcello.iriti@unimi.it (M.I.); 6 Institute of Fermentation Technology and Microbiology, Lodz University of Technology, Wolczanska 171/173, 90-924 Lodz, Poland; dorota.kregiel@p.lodz.pl (D.K.); hubert.antolak@gmail.com (H.A.); 7 Department of Medical Parasitology, Zabol University of Medical Sciences, Zabol 61663335, Iran; 8 Department of Food Science, Faculty of Food Engineering (FEA), University of Campinas (UNICAMP), Campinas 13083-862, Sao Paulo, Brazil; ncirone@unicamp.br; 9 Department of Botany, Lahore College for Women University, Jail Road, Lahore 54000, Pakistan; mussabuswaeshal@hotmail.com; 10 Department of Nutrition and Dietetics, Faculty of Pharmacy, University of Concepcion, Concepcion 4070386, VIII-Bio Bio Region, Chile; 11 Department of Pharmacy, University “G. d’Annunzio” of Chieti-Pescara, 66100 Chieti, Italy; 12 Phytochemistry Research Center, Shahid Beheshti University of Medical Sciences, Tehran 11369, Iran; 13 Department of Chemistry, Richardson College for the Environmental Science Complex, The University of Winnipeg, Winnipeg, MB R3B 2G3, Canada

**Keywords:** *Tagetes patula*, *Tagetes erecta*, *Tagetes minuta*, *Tagetes lucida*, Asteraceae, ethnopharmacology, antimicrobial

## Abstract

*Tagetes* (marigold) is native to America, and its cultivation currently extends to other countries in Africa, Asia, and Europe. Many species of this genus, such as *T. minuta*, *T. erecta*, *T. patula*, and *T. tenuifolia*, are cultivated as ornamental plants and studied for their medicinal properties on the basis of their use in folk medicine. Different parts of the *Tagetes* species are used as remedies to treat various health problems, including dental, stomach, intestinal, emotional, and nervous disorders, as well as muscular pain, across the world. Furthermore, these plants are studied in the field of agriculture for their fungicidal, bactericidal, and insecticidal activities. The phytochemical composition of the extracts of different *Tagetes* species parts are reported in this work. These compounds exhibit antioxidant, antiinflammatory, and enzyme inhibitory properties. Cultivation and the factors affecting the chemical composition of *Tagetes* species are also covered. In the current work, available literature on *Tagetes* species in traditional medicine, their application as a food preservative, and their antimicrobial activities are reviewed.

## 1. Introduction

The human species used plants for treating diseases throughout history. The therapeutic properties and efficacy of each plant were mainly based on popular observations. This approach significantly contributed to their prescription, even if their chemical constituents were not always completely known [1,2,3,4,5]. Commercial drugs substituted medicinal plants; however, worldwide, people still use natural products for primary healthcare [6,7,8,9,10].

Natural products proved their importance as sources of substances with therapeutic potential [2,6,11,12,13,14,15,16,17,18,19]. Plant medicines have some advantages, because their components act on different molecular targets, incorporating lower cost and fewer side effects [2,16,20,21,22,23,24,25,26,27,28,29].

*Tagetes* (marigold) is an important genus belonging to the Asteraceae family and consists at least of 56 species [30]. It is a plant which is native to America, but it is naturalized in other countries in Africa, Asia, and Europe [31,32]. *Tagetes* spp. can be cultivated as ornamental plants or can be found as wild species [33]. There are many species of this genus, such as *T. minuta*, *T. erecta*, *T. patula*, and *T. tenuifolia*, that are studied because of their application in the field of agriculture, where they exhibit fungicidal, bactericidal, and insecticidal activities, as well as anticancer properties [34,35,36], resulting in their exploitation as beverages and condiments in folk medicine [37,38].

Marigold extracts are characterized by the presence of diverse compounds with different properties, namely phenylpropanoids, carotenoids, flavonoids, thiophenes, and others [39]. It is known that the compounds produced by plants could vary based on many factors, including the part of the plant from which it is extracted, harvesting seasons, plant development stage, and geographical sources [39,40].

*T. patula* presented antioxidant and cytotoxic activity in previous studies; however, the extract did not display good results for antimicrobial action against fungi (*Trichophyton rubrum*, *Trichophyton mentagrophytes*, *Microsporum canis*, *Microsporum anisopliae*, and *Beauveria bassiana*) [41]. Conversely, *T. minuta* essential oil (EO) showed inhibition against *Aspergillus niger* and *Candida albicans* in addition to Gram-positive bacteria [42]. *T. erecta* showed that its compounds have antiinflammatory potential [20] and have anticancer activity [43], as well as *T. minuta*, which also presented cytotoxic [42] and antiinflammatory activities.

Collectively, the studies showed the potential of *Tagetes* spp. as alternatives to a wide variety of drugs. The many alternative uses for *Tagetes* spp. resulted in the depletion of natural sources because its extracts and specific metabolites have such a high demand. Thus, the crop needs systematic cultivation in tropical, subtropical, and temperate agroclimatic zones worldwide [44,45]. Marotti et al. [39] evidenced that most *Tagetes* spp. could be cultivated in environmental conditions different from those typical of the genus.

## 2. *Tagetes* Genus Plant Cultivation

*Tagetes* spp. are used in different areas for various purposes such as cosmetic preparation, medicine, and ornamentals. They are found in different colors and fragrances [46]. *Tagetes* species grow in the temperate forests and mountainous regions of most countries in the world. They originated in South America and spread throughout the world as weeds. It was reported that South Africa, Australia, Nigeria, India, Uruguay, Brazil, France, Chile, Bolivia, France, Kenya, and Argentina and are the main producing countries of *Tagetes* oil in the world. In India, *Tagetes* is found in the western Himalayas between altitudes of 1000 and 2500 m [47]. Himachal Pradesh and the hills of Uttar Pradesh are the main growing regions where *Tagetes* species occur in their natural habitat. The wild growth of *T. minuta* in these regions forms the most important source of “*Tagetes* oil” in India [45].

Mild climates are most suitable for the luxuriant growth of *Tagetes* species. Flowering in these species greatly improves during mild climates in temperatures ranging from 14.5–28.6 °C. However, higher temperatures (26.2–36.4 °C) adversely affect flower production. The species of *Tagetes* successfully adapted to different soil conditions. However, the most desirable conditions for enhanced production are a deep, fertile, friable soil having good water-holding capacity, being well drained with a pH close to 7.0–7.5. Sandy loam is ideal for the cultivation of *Tagetes* spp. The crop of *Tagetes* is suitable for cultivation in the plains, as well as on the hills, as a monocrop or intercrop in orchards/forest aromatic trees or as widely spaced crops such as rose-scented geranium. It is amenable for integration with traditional agricultural or aromatic crops in suitable field rotations. Cool temperatures induce germination, whereas high temperatures encourage profuse vegetative growth and flowering. Direct seeding (2.0–2.5 kg seeds/ha) or transplanting of 100–200-mm-long 30–60-day-old seedlings (0.50–0.75 kg seeds/ha for raising the nursery) with (300–600) × (150–300) mm spacing is practiced. A closer spacing of 300 × (150–300) mm for direct broadcasted or line-sown seeded crops and a wider spacing of (450–600) × 300 mm for transplanted crops are recommended. In less fertile soils, a closer spacing of 300 × 300 mm is suggested. Seeds germinate in 7–10 days [48]. Young seedlings are susceptible to weed competition. Two to three manual weeding operations are necessary until the crop establishes. Fully grown plants can smother the weeds. Nipping apical buds 50–60 days after seed sowing or 30–45 days after transplanting promotes the growth of branches and produces a crop canopy with a higher proportion of leaves and flowers. The crop can withstand short periods of moisture shortage. Phosphorus and potassium are usually applied basally, while nitrogen is applied in three equal splits upon planting, active vegetative, and flower bud initiation stages. The crop is occasionally affected by wilt (caused by *Sclerotiana sclerotium*, for which seed treatment with Thiram is suggested), *S. rolfsii* (seed treatment with Thiram is recommended), little leaf (caused by phytoplasma, for which spraying with *Streptomycin* is useful), marigold mosaic (caused by virus, infected plants are burnt) diseases, and collar rot (caused by *S. sclerotium*). Three to five irrigations at depths of 25–30 mm or at 0.5 IW:CPE ratio of irrigation scheduling [45] are sufficient for raising a good crop. The crop responds to applications of 20–30 tons of Farm Yard Manures (FYM), 100–200 kg N, 60 kg P_2_Os, 60 kg K_2_O/ha. Wild marigold is harvested manually with sickles during full flowering [49,50] (4–7 months duration) or seed setting [47,50] stages 200–300 mm above ground level. Crop duration is short (main 10 days, ratoon 30–60 days) in south India irrespective of whether the crop is planted in rainy (July/September) or winter (December) seasons [45,49]. In the rainy seasons, weeds become the major problem for the yield of marigold. If the weeds are not removed in time, great loss occurs in terms of growth and productivity.

## 3. Phytochemical Composition of EOs Obtained from *Tagetes* Genus Plants

The *Tagetes* genus is notorious for species rich in aromatic compounds and resinous exudate [51]. For 15 *Tagetes* species, it was possible to find information on the chemical composition of the EO contained in the aerial parts, capitula, or leaves.

**Figure 1:**
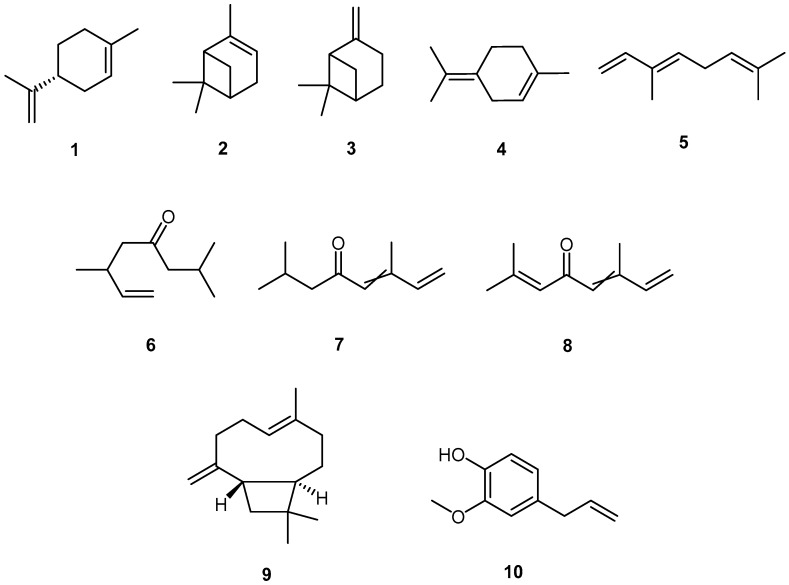
Main chemical structures found in the *Tagetes* essential oils (EOs): (**1**) limonene, (**2**) α-pinene, (**3**) β-pinene, (**4**) terpinolene, (**5**) (*E*)-β-ocimene, (**6**) dihydrotagetone, (**7**) tagetone (represented as a mixture of (*E*)- and (*Z*)-isomers), (**8**) tagetenone (represented as a mixture of (*E*)- and (*Z*)-isomers), (**9**) β-caryophyllene, and (**10**) eugenol.

Generally speaking, the oils are rich in monoterpene hydrocarbons (ocimenes, limonene, terpinene, myrcene, etc.) and in acyclic monoterpene ketones (tagetone, dihydrotagetone, and tagetenone) which are the primary odorants, in addition to lower amounts of sesquiterpene hydrocarbons and oxygenated compounds. Within these groups, the chemical diversity is quite high. The only striking differences in EO composition come from *T. lucida* and *T. filifolia*, whose EOs are dominated by phenylpropanoids such as methyleugenol, methylchavicol, and anethole. The EO from the leaves of *T. tenuifolia* contained 2.2% methylchavicol. Figure 1 presents the main chemical structures of the compounds found in the EOs, numbered for ease of reference in the other tables.

### 3.1. Components of T. patula

According to some taxonomical authorities [52,53], *T. patula* is to be considered a synonym of *T. erecta*. However, given the relative large amount of literature on its EO chemistry, where it is always considered as a separate entity, it is treated here as a close relative but a separate taxon from *T. erecta*.

#### 3.1.1. EOs from Aerial Parts (Comprising Capitula, Leaves, and Stems)

According to Tisserand and Young [54], the major components of the EOs from the aerial parts of the plants found in India, Egypt, and South Africa are summarized in Table 1.

Two recent reviews reported the chemistry of *T. patula* EOs [55,56] on the basis of the presence of (*Z*)-β-ocimene and (*E*)-β-ocimene, limonene, (*E*)-tagetone and (*Z*)-tagetone, methyl heptenol, β-caryophyllene, piperitone, piperitenone, α-terpinolene, (*Z*)- and (*E*)-tagetenones, (*Z*,*Z*)-alloocimene, and (*Z*)-β-ocimene epoxide.

The impact of the geographical origin on the chemical diversity was significant; an Indian *T. patula* EO contained limonene (13.6%) and α-terpinolene (11.2%), but not most typical compounds such as (*E*)-β-ocimene (8.3%), β-caryophyllene (8%), piperitone (6.1%), and piperitenone (4.9%) [57]. Another paper describing the composition of the EOs from Brazil [58] recognized the main components as 4-vinylguaiacol and γ-terpinene. Later studies found that the Brazilian EOs contained α-terpinolene, limonene, piperitenone, and piperitone as the main components [36].

#### 3.1.2. EOs from Capitula

The same reviews [55,56] found that capitula EOs contained limonene (2.1–6.2%), (*Z*)-β-ocimene (15–20%), α-terpinolene (7.8–15.6%), (*E*)-tagetone (1.4–2.5%), (*Z*)-tagetone (1.8–4.62%), piperitenone (8.1–22.9%), piperitone (10.6–24.7%), and β-caryophyllene (15.1–23.7%) as the main components.

There was high diversity in the composition, with certain EOs dominated by components which were scarce in others, such as isoborneol (3.5%), (*Z*)-tagetenone (12.4%), (*E*)-tagetenone (10.4%), pipertitenone oxide (5.8%), (*E*,*E*)-α-farnesene (2.5%), dihydrotagetone (4.9–6.2%), *p*-cymen-8-ol (11.0%), alloocimene (3.7%), and (*E*)-sesquisabinene hydrate (12.5%), while, in some instances, major compounds such as (*Z*)-β-ocimene or α-terpinolene were not present. Later studies found that capitula EO from from Venezuela [59] and from India [60] are dominated by the compounds listed in Table 2.

#### 3.1.3. EOs from Leaves

Leaf EOs usually contain limonene, (*Z*,*Z*)-alloocimene, (*Z*)-β-ocimene epoxide, (*E*)-tagetone, (*Z*)-tagetenone, piperitone, piperitenone, and α-terpinolene [55,56]. A later study found, however, that the main compounds are α-terpinolene (20.9%) and piperitenone (14.0%) [59].

### 3.2. Components of T. erecta

#### 3.2.1. EOs from Aerial Parts

Two previous reviews looked into the chemistry of *T. erecta* EOs [55,56] and found that the EOs from flowering aerial parts are characterized by dihydrotagetone, tagetones, tagetenones, piperitone, limonene, (*E*)-β-ocimene, linalyl acetate, linalol, terpinolene, *n*-nonyl aldehyde, β-phellandrene, piperitone, and β-caryophyllene, with minor compounds including thymol, carvacrol, indole, nerolidol, 1,8-cineole, tagetone, α- and β-pinenes, dipentene, menthol, and geraniol. One study [39] reported that Italian EOs contained dihydrotagetone, tagetones, tagetenones, and piperitone as the major compounds. A later study confirmed the presence of piperitone (35.9%) and terpinolene (22.2%) as major compounds in EOs from aerial parts in Venezuela [59].

#### 3.2.2. EOs from Capitula

According to previous reviews [55,56], the EOs from capitula contain limonene, ocimenes, linalyl acetate, linalol, tagetone, *n*-nonyl aldehyde, aromadendrene, phenylethyl alcohol, salicylaldehyde, phenylacetaldehyde, 2-hexen-1-al, eudesmol, tagetenone, myrcene, *p*-cymene, *d*-carvone, eugenol, terpinolene, (*Z*)-myroxide, piperitone, piperitenone, piperitenone oxide, and β-caryophyllene. Another paper relative to Italian EOs [39] found that the major compounds were piperitone (28.9%), terpinolene (5.8%), β-caryophyllene (3.8%), limonene (3.5%), linalol (2.7%), myrcene (1.8%), and terpinen-4-ol (1.1%).

#### 3.2.3. EOs from Leaves

Two articles [55,56] disclosed the components of leaf EOs as limonene, α-pinene, β-pinene, dipentene, β-phellandrene, linalol, geraniol, menthol, tagetone, nonanal, linalyl acetate, camphene, sabinene, myrcene, (*Z*)-β-ocimene, (*E*)-β-ocimene, γ-terpinene, terpinolene, *p*-mentha-1,3,8-triene, terpinen-4-ol, *p*-cymen-9-ol, piperitone, thymol, indole, carvacrol, piperitenone, geranyl acetate, β-elemene, cyperene, β-caryophyllene, (*E*)-β-farnesene, γ-muurolene, γ-elemene, and nerolidol.

Amongst the reviewed papers, one relative to Italian EOs [39] found that the major compounds were terpinolene (28.5%), piperitone (24.2%), limonene (15.6%), (*E*)-β-ocimene (4.7%), β-caryophyllene (2.0%), indole (1.4%), sabinene (1.1%), (*Z*)-β-ocimene (1.1%), and myrcene (1.0%); one relative to Chinese EOs from dried leaves [61] found terpinolene (37.9%), 2-isopropyl-5-methyl-3-cyclohexen-1-one (14.1%), limonene (13.1%), (*Z*)-β-ocimene (8.8%), β-caryophyllene (4.2%), (*E*)-β-ocimene (3.0%), *p*-mentha-1,3,8-triene (1.5%), γ-elemene (1.7%), and *p*-vinylanisole (1.1%) as the main components.

Another paper [62] reported the main components of leaf EOs from Brazil as piperitone (45.7%), limonene (9.7%), and piperitenone (5.9%). There were ambiguous data relative to the presence of phototoxic thiophenes in these plants. According to Marques and colleagues [62], a thiophene derivative, α-terthienyl, was present in the roots of *T. erecta*. This compound is able to generate singlet oxygen in organic solvents and superoxide radical anions in an aqueous medium. Gupta [55] mentioned the presence of β-terthienyl and α-terthienyl in *T. patula.* The data on the EOs from various parts of *T. erecta* are summarized in Table 3.

### 3.3. Components of T. minuta

The EOs from this plant and their compositions were elegantly reported in previous reviews [63,64,65,66,67].

#### 3.3.1. EOs from Aerial Parts

According to Tisserand and Young [54], the major components of EOs from the aerial parts of plants in India, South Africa, and Egypt are collected in Table 4.

According to Burfield [68], the major components of EOs from Madagascar are subject to considerable variation, since both ocimenes and dihydrotagetone can vary from 0.1% to 99%.

Two previous reviews explored the chemistry of *T. minuta* EOs [55,56] and identified (*Z*)-β-ocimene, dihydrotagetone, (*E*)-β-ocimene, (*Z*)- and (*E*)-tagetones, and (*Z*)- and (*E*)-tagetenones as the major components of the aerial parts. The (*E*)-β-ocimene content varied quite extensively across the studies. In one study, it was observed that drying the plant material prior to distillation changed the chemistry of the EO. Both EOs contained (*E*)-tagetenone, (*Z*)-β-ocimene, and (*Z*)-tagetone; however, the fresh plant contained larger amounts of (*Z*)-tagetenone, limonene, and allocimene, while the dried plant was characterized by a high content of dihydrotagetone.

According to Singh and colleagues [56], eight types of EOs from capitula or whole flowering plants can be recognized. These types were clustered into two larger supergroups, one made of EOs dominated by (*Z*)-β-ocimene, and a second made of EOs dominated by dihydrotagetone (Table 5).

The compositions of the EOs are, as shown, very variable. The major constituents of a commercial EO of *T. minuta* from Madagascar were determined by Juliani and coworkers [69] to be α-pinene (2.0%), limonene (7.4%), dihydrotagetone (11.6%), tagetone (14.1%), (*Z*)-tagetenone (6.7%), (*E*)-tagetenone (9.0%), β-caryophyllene (1.1%), and bicyclogermacrene (2.2%). Two EOs distilled from Indian plant material [30,70] are compared in Table 6.

Prakasa Rao and coworkers [49] examined the main EO constituents in Indian *T. minuta* plants harvested at various growth stages, and the results are summarized in Table 7.

In a comparative study of 18 different EOs from Madagascar [71], it was found that nine components were present with relatively high content, although with important variations in percentages. The compounds were limonene (3.6–11.0%), (*Z*)-β-ocimene (1.0–17.1%), (*E*)-β-ocimene (0.5–14.6%), *p*-cymene (0.3–20.4%), β-caryophyllene (1.1–12.7%), (*Z*)-tagetenone (traces (tr)–26.7%), (*E*)-tagetenone (tr–31.3%), α-muurolene (tr–36.5%), and verbenone (1.4–15.4%).

The authors concluded that there exist at least two different chemotypes, one characterized by a high content of terpenes such as limonene (10–13%), (*E*)-β-ocimene (0.5–14.6%), *p*-cymene (6–16.5%), and α-muurolene (11–28%), as well as trace amounts of tagetone derivatives, such as dihydrotagetone, tagetone, and tagetenone, with verbenone (5–15.4%) as the main oxygenated compound. Even within this chemotype, the variability could be very high, such as in the case of one sample characterized by a high content of caryophyllene oxide (5.5%), and α- and γ-cadinenes (4.5% and 9.5%, respectively). The presence of linalol (4.6%) seems to be a mistaken identification, since, according to Lawrence [67], this molecule is not present in *T. minuta* EO. The second proposed chemotype is characterized by a high content of tagetone derivatives with the sum of dihydrotagetone, (*E*)- and (*Z*)-tagetones, and (*E*)- and (*Z*)-tagetenones higher than 72%. It appears that these Malagasy EOs present a specific composition that differs from those described by Singh and colleagues [56], in that both (*Z*)-β-ocimene and dihydrotagetone are present at very low percentages.

A paper that examined a commercial sample of aerial part EOs from South Africa [72] found that the major compounds were (*Z*)-β-tagetenone (8.7%), (*E*)-β-tagetenone (6.9%), (*Z*)-tagetone (5.1%), allocimene (4.5%), and ethyl 2-methylbutanoate (1.0%). The most important molecules in terms of their odorant impact (a combination of their percentage in the EO and their olfactory threshold) were (*E*)-tagetenone, ethyl isobutyrate, 3-methyl-2-buten-1-thiol, ethyl isobutyrate, 2-methylfuran-3-thiol, decanal, linalol, terpinen-4-ol, (*Z*)-tagetenone, (*Z*)-tagetone, (*E*)-tagetone, alloocimene isomer, octanal, ethyl 2-methylbutyrate, octyl acetate, 1-nonen-3-ol, myrcene, 1-octen-3-one, methyl 2-methylbutyrate, hexanal, and butanone isomer.

A South African EO was dominated by ocimenes (45.0%), but also had an important percentage of 3-methyl-2-(2-methyl-2-butenyl)-furan (11.9%) [73].

EOs produced from plants growing in Argentina [74] had limonene (66.3%), (*E*)-tagetenone (19.1%), β-caryophyllene (14.8%), α-pinene (11.8%), (*Z*)-tagetenone (2.7%), α-humulene (1.4%), τ-cadinol (0.8%), germacrene D (0.4%), β-eudesmol (0.4%), and carvone (0.1%) as the main components.

An EO obtained from Brazilian plant material [75] contained the following major constituents: limonene (7.0%), (*Z*)-β-ocimene (5.1%), dihydrotagetone (54.2%), and (*E*)-tagetone (6.7%). A second Brazilian EO was dominated by piperitone (86.3%) and limonene (13.7%) [76]. Two EOs from Kenya were analyzed [77,78], and the results are summarized in Table 8.

A third Kenyan EO had (*E*)-tagetone, dihydrotagetone, and alloocimene as its main components, but also contained sabinene, α-phellandrene, limonene, (*Z*)-β-ocimene, (*E*)-β-ocimene, (*Z*)-tagetone, (*Z*)-tagetenone, (*E*)-tagetenone, elixene, silphiperfol-6-ene, (*E*)-caryophyllene, α-humulene, and bicyclogermacrene [79].

Another recent paper examined a commercial EO from the United States of America, which, although dominated by β-ocimene (36.4%), showed an uncharacteristically high content of limonene (26.9%), as well as high (*Z*)-tagetone content (16.9%), also observed in Rwanda EOs, in addition to a relatively high content of alloocimene (6.3%) and β-caryophyllene (4.5%), and relatively low amounts of (*Z*)-tagetenone (0.8%) and (*E*)-tagetenone (0.6%) [80].

The chemical diversity could be independent of geographical origin, but could depend on plant phenology. A paper on an Indian EO revealed that EOs from plants harvested in the winter (December–January) were dominated by ocimenes (just like the first group above), and those from plants harvested in autumn (October–November) were dominated by dihydrotagetone (just like the second group above), while plants harvested in the summer (June) were dominated by tagetone, and a tagetenone-rich oil was obtained in the winter if the seeds were sown in September [45].

#### 3.3.2. EOs from Capitula

Two previous reviews [55,56] proposed the main components of EOs from capitula of *T. minuta* as the compounds listed in Table 9.

Two papers examined various accessions of capitula EOs, as summarized in Table 10 [81,82].

Notable points in the composition of the EOs analyzed by Gila and colleagues [81] were the very high amount of (*E*)-β-ocimene, and the huge quantities of α-phellandrene, as well as the absence of (*Z*)-tagetone and (*E*)-tagetone.

Some papers evaluated the variation in EO content according to phenological stage. According to Worku and Bertoldi [83], who observed the evolution of EO composition from the pre-flowering stage to the immature seed stage, the content of (*Z*)-β-ocimene increased through the process from 7.2% to 37.5%, and the content of (*Z*)-tagetenone declined from almost 40% to 13.1%, while little difference was observed for (*Z*)- and (*E*)-tagetone. Chalchat and coworkers [84], measuring the same variations, found that the content of (*Z*)-β-ocimene increased dramatically from the pre-flowering (2%) to early flowering (20.4%) stage, and then decreased slightly. The content of (*Z*)-tagetenone increased from 0.1% to 8.3% at the early flowering stage, and then decreased again. Dihydrotagetone increased from 16.5% to 33.4%, (*E*)-tagetone decreased from 16.9% to 3.7%, (*Z*)-tagetone remained stable at levels of 18.2–23.4%, and (*E*)-tagetenone was stable around 0.2% apart from a peak at the early flowering stage of 2.2%.

Looking at the same variations, Lawrence [85] reported different results. Dihydrotagetone was the major compound and it decreased in content from 51% to 14%; (*Z*)-β-ocimene was the second most common compound, and its content increased from the pre-flowering to the post-flowering stage from 16.9% to 45.9%. The content of (*Z*)-tagetone saw a dramatic decrease from the pre-flowering (18.5–22.4%) to the early flowering (1.3%) stage, before a steep increase to 16.9–20.4%, while the content of (*E*)-tagetone varied between 1.4 and 2.1%. On the other hand, the (*Z*)-tagetenone content was variable with higher values in the early and full flowering stages (8.6%) and a lower content at the seed stage (0.3–0.4%), while (*E*)-tagetenone was stable at 1.5–3.6%.

A more recent paper [86], examining Iranian EOs at budding, full flowering, and seed set stages, found a reduced yield, from 1.0% to 1.6%. It also found that limonene was the main component at the budding stage, at 49.2%; however, its content decreased dramatically to 4.0% and then to 2.8%. Dihydrotagetone remained pretty stable during the changes, passing from 14.8% to 21.4% to 20.7%, while α-terpineol was also present in significant quantities, and its percentage increased from 7.4% to 15.6% to 18.4%. Moreover, (*Z*)-tagetone increased from 4.7% to 13.4%, (*Z*)-β-ocimene went from 4.4% to 8.3% at the flowering stage and decreased again at the seed stage to 7.4%, while (*Z*)-tagetenone content was stable around 3.1–4.5%, and (*E*)-tagetenone content increased from 3.3% to 11.8% at the flowering stage and decreased to 8.6% at the seed stage. Finally, spathulenol increased from 0.9% to 5.6%. Two papers analyzed the variations in EO content relative to various agronomical parameters [87,88], and they are presented in Table 11.

#### 3.3.3. EOs from Leaves

The review by Gupta and coworkers [55] found that the EOs from leaves were dominated by dihydrotagetone (2.7–54.2%), (*Z*)-β-ocimene (1.4–16.1%), (*E*)-tagetenone (0.1–19.5%), (*Z*)-tagetenone (tr–31.4%), (*E*)-tagetone (0.8–14.5%), (*Z*)-tagetone (6.6–28.2%), limonene (2–12.4%), eugenol (16.5%), isobornyl acetate (0.4%), *p*-menth-4-en-3-one (0.1%), sabinene (0.6–1.1%), and terpinen-4-ol (1.3%).

According to Singh and coworkers [56], leaf EOs are “rich in the distal compounds of the dihydrotagetone biosynthetic pathway which proceeds in the direction of (*Z*)-β-ocimene, (*E*)- and (*Z*)-tagetenone, (*E*)-and (*Z*)-tagetone, and dihydrotagetone”; five groups of leaf EOs can be recognized, classified according to geographical origins (Table 12).

A recent paper on Argentinian EOs [81] found that the composition did not fit in any of the proposed subgroups by Singh and coworkers [56], since the main molecule was dihydrotagetone (2.5–65.8%), while the next two most prominent molecules were α-phellandrene (31.0%) and (*E*)-β-ocimene (17.7%) (which never occurred as characterizing constituents found by Singh and coworkers [56]), followed by *o*-cymene (16.0%), (*E*)-tagetenone (0.8–30.7%), (*Z*)-tagetone (6.8–13.2%), limonene (4.0–10.4%), (*Z*)-β-ocimene (3.5–14.9%), (*Z*)-tagetenone (1.6–7.4%), and (*E*)-tagetone (1.2–7.8%). The authors identified three chemotypes, one having (*Z*)-β-ocimene, dihydrotagetone, (*Z*)-tagetone, (*E*) and (*Z*)-tagetenone, and limonene as the major constituents; the second containing mainly dihydrotagetone; and the third characterized by a high percentage of α-phellandrene and (*E*)-β-ocimene. While the first two chemotypes are recognizable in the subdivisions proposed by Singh and coworkers [56], the third is not.

Another EO from Egypt [89] was dominated by (*E*)- and (*Z*)-tagetone, which together accounted for 52.3–64.2% of the EO, which also contained limonene (18.2%), spathulenol (6.9%), dihydrotagetone (5.9%), linalol (5.9%), α-gurjunene (2.3%), sabinene (2.3%), longifolene (2.2%), terpinen-4-ol (1.4%), and β-caryophyllene (1.2%). On the other hand, an EO from Yemen [90] was characterized by (*Z*)-tagetenone (15.9%) and (*E*)-tagetenone (34.8%), and secondarily by (*Z*)-β-ocimene (8.3%), limonene (2.3%), (*Z*)-tagetone (1.8%), dihydrotagetone (1.4%), and a dimethylvinylketone derivative (20.6%).

An EO distilled from Iranian plant material [42,91] contained dihydrotagetone (33.9%), (*E*)-tagetenone (19.9%), (*E*)-β-ocimene (19.9%), (*E*)-tagetone (16.1%), (*Z*)-β-ocimene (7.9%), (*Z*)-tagetenone (5.3%), limonene (3.1%), (*E*,*Z*)-epoxy-β-ocimene (2.0%), *p*-cymene (0.9%), (*Z*)-isoeugenol (0.9%), thymol (0.5%), carvacrol (0.5%), alloocimene (0.4%), sabinene (0.4%), germacrene D (0.4%), spathulenol (0.4%), α-pinene (0.3%), β-caryophyllene (0.3%), (*Z*)-3-hexenyl acetate (0.2%), (*Z*)-tagetone (0.2%), and α-humulene (0.2%).

Moreover, two papers analyzed the variations in EO content relative to various agronomical parameters [87,88], and they are presented in Table 13.

#### 3.3.4. EO from Fruits

According to Gupta and Vasudeva [55], mature fruits with seeds contained (*Z*)-β-ocimene (6.4–36.8%), (*Z*)-tagetone (10.5–17.1%), (*Z*)-tagetenone (0.5–3.0%), and (*E*)-tagetenone (0.2–7.5%), in addition to dihydrotagetone (35.7%), (*E*)-β-ocimene (15.5%), limonene (5.4%), β-phellandrene (4.7%), and sabinene (0.2%). In addition, *T. minuta* could present trace amounts of α-terthienyl.

### 3.4. Components of T. lucida

#### 3.4.1. EOs from Aerial Parts

While *Tagetes* species are generally characterized by the content of tagetones, tagetenones, etc., *T. lucida* EOs from aerial parts mainly contain phenylpropenes and terpenes [55]. In fact, according to Ciccio [92] and Marotti and coworkers [39], the EO is dominated by methyl chavicol (estragol) at levels up to 97.3%. However, according to other authors [68,93,94], at least four chemotypes can exist, characterized by the main presence of (a) high levels of (*E*)-anethole (up to 74%) and low to very low levels of methyl chavicol (11.57%) or methyleugenol (1.8%), and germacrene D; (b) high levels of methyl chavicol (up to 97%), in addition to methyleugenol, methylisoeugenol, and germacrene D; (c) high levels of methyl eugenol (up to 80%), in addition to methylchavicol and methylisoeugenol; and (d) high amounts of nerolidol (around 40%), in addition to methyleugenol, methylchavicol, and caryophyllene oxide. Other compounds include linalol (0.3–3.7%), myrcene (1.3%), (*E*)-β-ocimene (2.9%), linalol (1.1%), β-caryophyllene (0.5–2.4%), germacrene D (tr–5.4%), methylisoeugenol (tr–3.9%), bicyclogermacrene (0.6%), spathulenol (tr–0.2%), and caryophyllene oxide (tr–10.3%). These data were confirmed by later papers; an EO distilled from aerial parts from Colombia [95,96] displayed methylchavicol (92.1%), β-myrcene (5.9%), (*E*)-β-ocimene (1.3%), and linalol (0.3%), while an EO from Egypt [97] contained over 90% methyl chavicol.

#### 3.4.2. EOs from Leaves

An EO distilled from Italian plant material [39] contained methyl chavicol (78.2%), methyl eugenol (3.6%), and β-caryophyllene (9.4%).

#### 3.4.3. EOs from Flowers

An EO distilled from Italian plant material [39] was characterized by methyl chavicol (93.8%), methyl eugenol (0.1%), and β-caryophyllene (2.1%).

#### 3.4.4. Other Compounds

According to Ciccio [92], *T. lucida* could show small amounts of α-terthienyl.

### 3.5. Components of T. filifolia

According to Gupta and Vasudeva [55], EOs from aerial parts showed high amounts of (*E*)-anethole (76.9–87.5%) and methylchavicol (10.7–19.3%), in addition to variable amounts of (*Z*)-anethole (tr–68.2%), and lower amounts of isomenthone (4.5%), menthone (4%), 1,8-cineole (1.5%), pulegone (1.1%), germacrene D (1%), bicyclogermacrene (1%), (*E*,*E*)-α-farnesene (0.8%), cumin aldehyde (0.7%), and spathulenol (0.5%). An EO from Argentina [98] was characterized by only two molecules: (*E*)-anethole (74.5%) and methylchavicol (23.7%). This chemical composition is uncharacteristic of *Tagetes* species, and is close to the specific chemotype of *T. lucida*, rich in (*E*)-anethole.

### 3.6. Components of T. terniflora

According to Gupta [55], EOs from aerial parts presented (*Z*)-tagetone (31.0%), (*Z*)-β-οcimene (15.4%), (*E*)-tagetenone (15.4%), (*Z*)-tagetenone (14.5%), (*E*)-tagetone (10.3%), and dihydrotagetone (6.5%) as the main components, in addition to (*E*)-β-ocimene, limonene, isomenthone, spathulenol, (*Z*)-anethole, and (*E*)-anethole. An EO from leaves produced in Argentina [99,100] showed a similar composition, with (*E*)-β-ocimene (27.3%), (*Z*)- and (*E*)-tagetone (26.0%), (*Z*)- and (*E*)-tagetenone (17.5%), and dihydrotagetone (16.8%).

### 3.7. Components of T. tenuifolia

An earlier paper [101], examining two EOs from aerial parts, registered the following main components: (*Z*)-ocimenone (9.1–26.3%), (*E*)-ocimenone (9.6–26.3%), dihydrotagetone (13.4–17.3%), tagetones (5.5–12.9%), limonene (8.7–10.2%), and β-ocimene (tr–6.0%). Of particular interest was the presence in one EO of significant quantities of thujone (11.9%). According to the recent review by Gupta [55], the EOs are characterized by dihydrotagetone, tagetones, ocimenones, and piperitone. One of the review papers [39] gave a breakdown of the composition of leaves and flowers, which were fairly consistent, with (*E*)-tagetenone, dihydrotagetone, (*E*)-tagetone, and (*Z*)-β-ocimene as the four main components. The only notable difference was that the leaf EOs contained 2.2% methylchavicol, while the flowers contained 2.0% camphor.

### 3.8. Components of T. mandonii

According to Gupta [55], EOs from aerial parts were characterized by (*Z*)-β-ocimene, (*E*)-ocimene, tagetenones, tagetones, limonene, spathulenol, and (*Z*)-anethole. According to an older paper that analyzed the EO of *T. maxima*, which is now recognized as a synonym of *T. mandonii* [102], the composition was dominated by (*Z*)-tagetone (31.3%), dihydrotagetone (26.7%), and (*E*)-tagetenone (22.4%), whereas other minor compounds comprised (*Z*)-tagetenone (5.4%), (*E*)-tagetone (2.8%), methyl eugenol (1.0%), (*Z*)-β-ocimene (1.0%), *p*-menth-4-en-3-one (1.0%), β-caryophyllene (0.3%), (*E*)-myroxyde (0.3%), germacrene D (0.2%), (*Z*)-myroxyde (0.2%), (*E*)-β-ocimene (0.2%), limonene (0.2%), 1,8-cineole (0.2%), α-humulene (0.1%), and sabinene (0.1%).

### 3.9. Components of T. multiflora

According to Pichette and coworkers [102], EOs from aerial parts had (*Z*)-tagetone (47.3%), (*E*)-tagetenone (17.2%), and (*Z*)-β-ocimene (12.8%) as the main components, and dihydrotagetone (8.1%), (*Z*)-tagetenone (3.5%), (*E*)-tagetone (1.5%), α-phellandrene (0.7%), β-caryophyllene (0.7%), *p*-menth-4-en-3-one (0.7%), α-humulene (0.3%), 1,8-cineole (0.2%), germacrene D (0.2%), sabinene (0.1%), (*E*)-β-ocimene (0.1%), and (*Z*)-myroxyde (0.1%) as minor compounds.

### 3.10. Components of T. lemmonii

The flowering stems of *T. lemmonii* were rich in ethyl-2-methyl butyrate (0.3%), α-phellandrene (0.2%), (*E*)-β-ocimene (2.1%), dihydrotagetone (42.5%), alloocimene (2.8%), (*Z*)-tagetone (0.04%), (*E*)-tagetone (16.1%), β-caryophyllene (0.2%), (*Z*)-tagetenone (3.9%), (*E*)-tagetenone (14.2%), and germacrene D (0.5%) [103].

### 3.11. Components of T. rupestris

The EO from *T. rupestris* (Argentina) contained (*Z*)- and (*E*)-ocimenes, (*Z*)- and (*E*)-tagetones, and (*Z*)- and (*E*)-tagetenones as the major compounds [104].

### 3.12. Components of T. subulata

The capitula and leaves of *T. subulata* were characterized by terpinolene (26.0%), piperitenone (13.1%), and limonene (10.8%) [59].

### 3.13. Components of T. caracasana

The EOs from *Tagetes caracasana* (Venezuela) contained (*E*)- (64.3%) and (*Z*)-tagetone (13.7%) as the main compounds [59].

### 3.14. Components of T. pusilla

The EO of the leaves of *T. pusilla* from Venezuela contained (*E*)-anethole (70%) and 4-allylanisole (30.0%) as the main compounds, although, in EOs from Bolivia, the only observable compounds were (*E*)-anethole (92.2%) and α-pinene (0.4%) [105].

### 3.15. Components of T. mendocina

The EO distilled from plant material from Argentina contained (*E*)-β-ocimene, (*Z*)-tagetone, (*E*)-tagetone, (*Z*)-ocimenone, α-pinene, and (*E*)-ocimenone as the main components (>3.5%) [106].

## 4. Traditional Medicine Uses of *Tagetes* Genus: Ethnopharmacological Relevance

Traditionally, different parts of some *Tagetes* species are used as remedies to treat various health problems across the world. In Bangladesh, the leaves of *T. patula* are applied on boils and carbuncles and used against kidney troubles, muscular pains, and piles. Their juice is prescribed for earache and opthalmia [107]. In Pakistan, both leaves and flowers are collected and used as an antipyretic [108]. Other uses were recorded for *T. filifolia* in Mexico, where the Pima tribe prescribes a cup of tea prepared with its branches for stomachache [109], and in Argentina, where it is recommended for infected wounds [110]. Wounds and sores are also healed with leaf and flower decoctions or infusions of *T. minuta* [111], while a topical application of its sap is used in Kenya [112]. A particular use of *T. minuta* for wound healing in dental disorders was reported by Rahman et al. [113]. Ata and coworkers [114] attributed a general use in skin diseases. In Argentina, Bolivia, Brazil, Paraguay, and Peru, *T. minuta* infusions and decoctions are considered as digestives, appetizers, cholagogues, carminatives, gastric sedatives, antidiarrheics, and vermifuges. They are administered against food poisoning as antiparasitics and to cure dyspepsia, gastritis, intestinal colic, and flatulence, while the chewed fresh leaves are recommended for removing bad breath. The leaf decoction is prepared as an expectorant or an antiabortive, and is also used in order to reduce milk secretion. The infusion regulates menstrual flow and is used for vaginal washes in cases of infected flows. The whole plant is a febrifuge and diuretic [111]. *T. minuta* leaf and flower infusions are now incorporated in home medicines of the descendants of Polish migrants in Argentina as a prophylaxis after labor [115]. Ijaz and coworkers [116] documented new Pakistani uses of *T. minuta* leaves against cough and stomach disorders. Furthermore, their use against children’s cough (three decoction teaspoons thrice per day for a week) and headache is rooted in Southern Uganda [117]. Both in new and old world countries, the leaves or the entire plant are indicated for liver diseases through internal (tea or juice) or external (poultice) administration [118]. Although scarcely used, leaves and stems without sap are externally applied in Turkey for earache [119], while flower tea is drunk for musculoskeletal ailments in Morocco [120]. *T. minuta* and *T. lucida* are recommended for treating emotional and nervous disorders as part of a mixture with other anxiolytic plants. In Bolivia, the infusion of the *T. minuta* is used as a tonic for nerves [111], while, in Brazil, it is used as a sedative to drink before sleeping [121]. *T. lucida*, known to the Aztecs as a remedy for fever, diuresis, and epilepsy, was also used to treat tumors and age-related brain disorders such as dementia and fear [122,123]. It is sold instead of *Hypericum perforatum* in different regions of Mexico, where the aerial parts are still consumed orally in infusions and hydroalcoholic extracts to soothe anxiety, depression, irritability, and sadness [124,125]. Mexican traditional medicine prescribes *T. lucida* for “nervios” and “susto”, two culture-bound syndromes described as illnesses characterized by a “state of bodily and mental unrest” able to decrease the ability to achieve daily goals and as a condition of being frightened and “chronic somatic suffering stemming from emotional trauma”, respectively [124,125]. There are several minor uses for the treatment of gastrointestinal, respiratory, and urogenital systems, and against rheumatism, ulcers, and inflammation. Moreover, *T. lucida* is recommended as a stimulant of the immune system and decoctions of its aerial parts fight infections caused by some helminthes and protozoa (e.g., ascaridiasis and giardiasis) [123,124,125,126]. *T. lucida*, together with *T. erecta* and *T. tenuifolia*, is an important plant for treating folk illnesses considered cold or “friadad” such as “frío en el estómago” (cold in the stomach), “calor en el estómago” (heat in the stomach), and “empacho” (indigestion), as well as constipation, baby and child diarrhea, and eye irritation [127]. The remedies prepared with aerial parts of these plants are ointments employed in topical administration, in seat baths and in specific sweat lodges, in addition to flower infusions and tinctures consumed orally [127,128]. The use of *T. erecta* was documented in phytomedicine from Guatemala to cure the respiratory system against pneumonia, asthma, and tuberculosis, to cope with colic, for use as an antibiotic, analgesic, and antileukemic, and for wound healing, and immune system stimulating, as well as against headache, tetanus, and various parasites [129]. The efficacy of *T. erecta* on parasitic infections, as well as in the puerperium, is increased by combining all its aerial parts with those from other aromatic species in a Mexican syrup. Only the petals, however, are collected for the preparation of remedies useful in the affections of the central nervous system [128]. The infusion of *T. erecta* flowers covers a wide range of other actions against flu, fever, body pain, rash, sore throat, heart attack, and arthritis [130]. Among plants collected for medical purposes in India, *T. erecta* flowers are claimed to treat several skin diseases (sores, wounds, burns, ulcers, eczema, boils, and carbuncles), as well as earache, piles, and muscular pains [131]. Its extract—two teaspoons twice daily for 8–10 days—combined with common salt and minerals treats kidney problems, specifically removing blocked urine [131,132]. The leaves are used to relieve pain and remove inflammation [133]. *T. erecta* is used in Spanish and French herbal medicine as an external detersive, resolutive, and vesicant [134]. The inhabitants of Madagascar recognize that *T. erecta* has antimalarial properties, while the people of Rodrigues Island cure fever due to infection by drinking one cup per day of an infusion of three flowers [135,136]. Mauritians suggest a glass of *T. lucida* flower decoction in the case of abdominal pain related to diseases of the circulatory system, and in the case of neonatal jaundice for breastfeeding mothers [137]. Indian folk veterinary medicine applies drops of *T. erecta* flower extract thrice a day to cows and buffalos for otitis [138], and applies leaves to limit bleeding and to cure broken horns, external injury, and eye diseases [139]. In southern Ethiopia, leaves and stems of *T. minuta* are chopped, mixed with water, and given orally to cattle and sheep affected by anthrax, blackleg, and amoebiasis [140].

## 5. Food Preservative Applications of *Tagetes* Genus Plants

Metabolites synthesized by plants belonging to the genus *Tagetes* show significant effects as antioxidants, enzyme inhibitors, precursors of toxic substances, and pigments. In addition, these bioactive compounds are involved in photosensitization and energy transfer, actions of plant growth hormones and regulators, control of respiration and photosynthesis, and defense against parasites, bacteria, fungi, and some insects. On the other hand, some of the secondary metabolites, especially those contained in the flowers, are responsible for the attraction of pollinators. It is believed that the activity of the secondary metabolites in the *Tagetes* species, like in the case in other plants, is related to their composition, concentration, and environmental conditions affecting their content. Thus, plant extracts or EOs obtained from different parts may show different biological abilities, and therefore, can be used in a variety of industries, including cosmetic, pharmaceutical, or food production [141,142]. Although the extracts from these plants are not so popularly used as preservatives, *Tagetes* spp. are characterized with high potential in the field of agriculture. According to the approach “from the field to the fork”, the use of *Tagetes* spp. as bioactive agents in plant protection against micro- and macro-organisms is directed toward the first stages of the production, mainly in the plant breeding or post-harvest stages.

## 6. *Tagetes* spp. as Potential Plants in Agriculture

One of the first papers on marigold and its potential agricultural uses was published in 1958 by Uhlenbroeck and Bijloo, who studied the nematicidal activity of extracts obtained from the roots [143]. The results of their research indicated that prepared formulations were active against *Heterodera rostochiensis*, *Ditylenchus dipsaci*, and *Anguina tritici*. In later years, Swarup and Sharma [144] showed that *T. erecta* root extracts showed cidal or inhibitory activities against other plant-pathogenic nematodes, *Meloidogyne javanica* and *M. arenaria*, while Munoz et al. [145] indicated the potential use of *T. halisciencis* roots against *M. incognita.* The results of the study were important due to the fact that these nematodes are major agricultural pests of a wide range of crops cultivated in tropical and subtropical regions, as well as in greenhouses in temperate climates. *T. patula* showed activities against *Caenorhabditis elegans* and *Pratylenchus penetrans*, which are nematodes known as worms of gardens, compost piles, and rooting fruits [146]. *Tagetes* spp. produce a number of potentially bioactive compounds; however, thiophene derivatives and particularly α-terthienyl derivatives seem to be responsible for these nematocidal properties [147].

In addition, extracts and EOs obtained from the *Tagetes* genus were reported as insecticidal and larvicidal natural agents. Generally, *T. erecta* showed insecticidal activity against *Tribolium* spp., which are considered to be common pests of cereal silos, mills, and warehouses. The presence of these insects in stored food directly affects its quantity and quality [148,149]. Extracts obtained from *T. erecta* showed good insecticidal properties against aphids (*Aphidoidea*) and fall armyworm (*Spodoptera frugiperda*), which are responsible for the destruction of wild varieties of crops [150,151]. According to dos Santos et al. [152,153], the species *T. erecta* and *T. patula* showed larvicidal and insecticidal properties with the possibility of the use of *Tagetes* extracts as an alternative to synthetic insecticides used against rice weevil (*Sitophilus zeamais*).

From another point of view, Zoubiri and Baaliouamer [154] documented the high effectiveness of EOs of *T. minuta* against mosquitoes of *Anopheles gambiae*, which are responsible for malaria transmission. The results were comparable to commercial, synthetic, insecticidal agents. What is more, *T. minuta* oil possesses phytotoxicity toward common weeds, including green amaranth (*Amaranthus viridis*), vegetable amaranth (*A. tricolor*), hairy beggars tick (*Bidens pilosa*), little seed canary grass (*Phalaris minor*), nettle leaf goosefoot (*Chenopodium murale*), and barnyard grass (*Echinochloa crus-galli*) [155]. Going further, *Tagetes* spp. are potential chromium hyperaccumulators. According to Coelho et al. [156], at Cr(III) concentrations up to 0.12 mmol/L, the plants accumulated levels above that proposed for hyperaccumulators while still maintaining considerable growth and even flourishing.

## 7. *Tagetes* spp. as Antimicrobial Agents

Despite the lack of a clear and direct reference of extracts and EOs from *Tagetes* spp. as food preservatives, the activity of compounds from *Tagetes* spp. was noted against various microorganisms (Table 14).

In research conducted by Tereschuk et al. [157], extracts obtained from leaves of *T. minuta* showed antimicrobial activity against both Gram-positive and Gram-negative bacteria. The major component of the extract, quercetagetin-7-arabinosyl-galactoside, showed significant antimicrobial activity against tested pathogen microorganisms. In studies conducted by Tereschuk et al. [158], almost all of the tested strains were susceptible to *T. terniflora* extracts at concentrations of 200 mg/mL, except for bacteria *Zymomonas mobilis* and *Lactobacillus plantarum*, and yeast *Saccharomyces cerevisiae*. Moreover, these results were generally comparable with chloramphenicol at 100 mg/mL. Hernández et al. [159] indicated that *T. lucida* extracts, containing 5,7,4′-trimethoxyflavone, showed wide antibacterial activity. *T. lucida* extracts also showed high activity against Gram-negative bacteria and phytopathogenic fungi in the study conducted by Cespedes et al. [160]. Antifungal activities of the EOs against species belonging to *Candida*, *Penicillium*, and *Aspergillus* were also described [161,162]. What is more, Gakuubi et al. [79] documented the antibacterial activity of *T. minuta* EOs against phytopathogenic bacteria *Pseudomonas savastanoi* pv. *phaseolicola*, *Xanthomonas axonopodis* pv. *phaseoli*, and *X. axonopodis* pv. *Manihotis*, which are responsible for different plant diseases. The results obtained confirmed the biopesticidal nature of EOs of *Tagetes* spp. and their potential uses as cheap, safe, and effective alternatives to chemical bactericides used for the protection of agricultural products. Recent research on the antimicrobial potential of *T. minuta* was done by dos Santos [141], correlating the extract of this plant with traditionally used antibacterial, insecticidal, biocide, disinfectant, anthelminthic, antifungal, and antiseptic agents.

The results indicated that *Tagetes* spp. may potentially play an important role in food preparation and food preservation, as well as for use as an excellent food spice. Even from a traditional point of view, the nature of *Tagetes* spp. and their composition affect the quantity and quality of the extracts [172]. Despite promising results obtained in vitro, more detailed studies of the mechanisms of action of *Tagetes* spp. extracts and EOs would be beneficial for reaching their potential in biotechnology. It was documented that EO components, especially terpenoids such as dihydrotagetones, tagetones, and ocimenones, are sufficient to account for the observed antimicrobial activity [171]. Further progress in determining the active components of the EOs may be achieved by fractionating the EOs of *Tagetes* spp. and determining the antimicrobial activity of each component individually. Caution is required in this approach, as both synergistic and antagonistic antimicrobial interactions between constituents of EOs can occur, giving the whole extract a different activity from the sum of the individual components.

## 8. *Tagetes* spp. as Functional Food Additives

The concept of the development of a functional food may involve the integration of health-promoting substances into a multifunctional ingredient, which could be used to design a wide range of novel food products [173,174,175]. *Tagetes* spp. present compounds such as flavonoids and carotenoids that are not part of the EOs but have several functions as biologically active compounds. For example, *Tagetes* spp. flavonoids were recognized as antiallergic, antiinflammatory, antiviral, antiproliferative, and anticarcinogenic substances. Infusions, tinctures, and juice from aerial parts of *Tagetes* spp. are used as traditional food additives worldwide. In turn, *T. terniflora* is a native plant known as “suico-suico” or “quichia”, widely used in Argentina as a condiment in soups [158,176]. *T. minuta* can be used in hot or cold refreshing beverages.

Current epidemiological studies indicate that high flavonoid consumption is associated with reduced risk of chronic diseases, such as cardiovascular diseases. The global publicity of traditional medicine during the past two decades, together with advances in ethnopharmacological knowledge, increased the interest in flavonoids and their interaction with mammalian cells and tissues. Leaf infusions from members of *Tagetes* are used in folk medicine in the treatment of stomach and intestinal diseases. An important factor is that EOs and extracts from *Tagetes* spp. show no antimicrobial activity against human microflora, regarded as central to human immunity, such as *Lactobacillus* species. An absence of antimicrobial activity against nonpathogenic human bacteria could be beneficial for intestinal disease treatments, in which the intestinal flora must be preserved [177].

*Tagetes* EOs are potential agents for protecting food crops on the farm and during storage, thereby increasing food security, particularly in undernourished communities of the world. These EOs also provide an opportunity for developing environmentally friendly and nontoxic acaricide agents to enhance increased production of milk, beef, and hides/skin in the livestock industry [172].

*Tagetes* species were originally used as a source of EOs, with applications as flavoring in the food industry, extracted from leaves, stalks, and flowers. What is more, marigold pigments have potential as a natural food colorant. The flowers and their extracts are rich in orange/yellow carotenoids [141]. The status of marigold as a source of natural carotenoids was reviewed by Verghese [178,179]. The stability of these pigments was studied for oil-in-water emulsions or arabic gums. It was concluded that the composition of the emulsifying agents and the pH level have important roles in determining the effectiveness of the emulsions against color loss and coalescence kinetics. Anaerobic and lactic acid treatments of fresh flowers are promising in terms of pigment stability. What is more, compared with other natural sources of yellow and orange color, like turmeric, chill, and saffron, marigold is a cheaper and easily available source. Factors influencing the qualitative and quantitative profiles of carotenoids in flowers of marigolds are their storage conditions. It is widely known that carotenoids are beneficial for human health. However, the biological functions of many individual carotenoids like zeaxanthin, cryptoxanthin, antheraxanthlin, and neoxanthin, which are present in a large amount in marigold flowers, are not known. Although marigold as a colorant offers a strong intense orange to yellow color, no toxicity data are available in the literature on marigold extract, whether partially purified or raw, which makes it unusable in food.

It is worth noting that marigold flowers can be a cheaper source as a starting material for the isolation of lutein, a valuable natural pigment that can also serve as a nutraceutical. The deficit of this compound in the human body is probably responsible for the age-related impairment of vision. Dried flowers of *T. erecta* contain 0.1–0.2% carotenoids in dry matter, out of which 80% are lutein diesters [180]. In *T. erecta* and *T. minuta*, lutein was isolated, identified, and approved by the Food and Agriculture Organization (FAO) and the European Union [181,182,183]. This bioactive compound can find application as a food colorant and flavor in various foodstuffs. Additionally, the orange pigment extracted from the petals of marigold is in great demand for poultry feed.

Another valuable compound in marigold is quercetagetin. The in vitro antioxidant activity of this flavonoid and its potential in the control of diabetes mellitus and obesity were investigated by Wang et al. [184] and compared to quercetin and rutin. It was documented that quercetagetin has potential antidiabetic and antilipemic activities, showing inhibitory effects of α-glucosidase, α-amylase, and pancreatic lipase. These results may be essential scientific support for the application of quercetagetin as a nutraceutical for the treatment of diabetes and obesity. What is more, according to the newest research, both flavonoids and carotenoids, primarily patuletin, constituents of *T. patula* extract can protect Jurkat cells (human T lymphoblastoid cell line) from hydrogen peroxide responsible for oxidative stress. These findings were presented by Chkhikvishvili et al. [185] and they are in line with the antioxidant and antiinflammatory properties of marigold preparations used in folk medicine.

## 9. Antimicrobial Ethnomedicine of the *Tagetes* Genus

Despite being a genus with a considerable number of species (more than 50, according to The Plant List [52]), only four species were found for the treatment of symptoms associated with bacterial and fungal diseases, namely *T. erecta*, *T. filifolia*, *T. lucida*, and *T. minuta* (Table 15). All plants are commonly indicated for diseases of the digestive system, and only *T. filifolia* is not indicated for the treatment of diseases of the skin and subcutaneous tissue. *T. erecta* and *T. lucida* are used against diseases of the respiratory system [186].

The use of these species crosses continents such as America, Asia, and Africa, and, according to registries, the whole plant or only parts of the plant can be used, with no reports found for the use of the root. The form of use can be both internal and/or external, and the plant is used from a raw to a cooked (decoction) state. In the scientific milieu, a constant need to validate popular knowledge through assays that seek to ascertain whether the medicinal plant has any influence on the growth of microbial populations is seen, whether reducing or decimating them. In the following sections, the results of research in which plants from the *Tagetes* genus were investigated for their effect on pathogenic bacteria and fungi are discussed.

## 10. Antibacterial Activity of Plants from the *Tagetes* Genus

Igwaran et al. [197] evaluated the properties of the EO from *T. minuta* flowers and confirmed its antibacterial activity through microdilution considering the minimum inhibitory concentration (MIC). The EO showed good antibacterial activity against all tested strains. The MIC of 0.06 mg/mL was shown for *Vibrio* spp., *E. coli*, *Enterobacter cloacae*, and *Listeria ivanovii*, while the MICs against *S. aureus*, *Mycobacterium smegmatis*, and *Streptococcus uberis* were higher (0.125 mg/mL). As a positive control, the antibiotic ciprofloxacin was used and showed activity against *M. smegmatis* (0.06 mg/mL), *E. coli* (0.125 mg/mL), and *Vibrio* spp. (0.25 mg/mL).

Lambrecht et al. [198] tested the EO and hydroalcoholic extract from the aerial parts of the plant through a broth microdilution assay to determine the MIC. Although all plant samples showed antimicrobial activity, the hydroalcoholic extracts showed the most satisfactory results with all bacterial species. The *T. minuta* EO showed antimicrobial activity against all Gram-positive bacteria with EO percentage activities of 1.3% for *S. aureus*, 0.6% for *Staphylococcus coagulase* (+), 2.5% for *S. coagulase* (−), and 0.8% for *S. uberis*. On the other hand, for Gram-negative bacteria, the percentage activity values were 4.0% for *P. aeruginosa* and 5.0% for *E. coli*.

Two species, *T. patula* and *T. erecta*, were evaluated by Ayub et al. [199], who observed the antimicrobial activity of the petal hexane and methanolic extracts by disc diffusion (100 μL of the extract) and microdilution (0.03 to 72.0 mg/mL). The tests demonstrated antibacterial activity against the *E. coli*, *Pastrulla multocida*, *B. subtilis*, and *S. aureus* strains, with *T. patula* presenting inhibition zones with values varying from 12.4 mm to 20.2 mm for the methanolic extract and 8.2 mm to 11.4 mm for the hexane extract. Meanwhile, the *T. erecta* species showed inhibition zones varying from 10.0 mm to 17.5 mm (methanolic extract) and 8.3 mm to 10.0 mm (hexane extract). The MICs for *T. patula* were from 0.19 mg/mL to 4.05 mg/mL (methanolic extract) and from 4.05 mg/mL to 24.0 mg (hexane extract), while *T. erecta* displayed MICs from 0.63 mg/mL to 7.6 mg/mL (methanolic extract) and from 6.8 mg/mL to 24.4 mg/mL (hexane extract).

Shahzadi and Shah [200] tested the antimicrobial activity of crude, aqueous, ethyl acetate and butanol *T. minuta* extracts, in addition to leaf and stem extracts extracted with ethyl acetate and butanol. The antimicrobial activity was determined from cavity diffusion (20 μL) against Gram-positive and Gram-negative strains. Inhibition halos of the crude extract were from 5 mm to 6.5 mm against *Pseudomonas picketti*, *S. aureus*, and *B. subtilis* strains. The aqueous extract had inhibition halos only against *B. subtilis* (5.7 mm). However, the ethyl acetate extract from the flowers and seeds presented activity against *S. aureus* (3.0 mm) and *P. picketti* (5.0 mm), while the butanolic extract was active against *P. picketti* and *B. subtilis* (5.2 mm and 7.6 mm, respectively). The inhibitory results from the leaf and stem extracts showed activity against *S. aureus* (3.1 mm), *P. picketti* (6.3 mm), and *B. subtilis* (6.1 mm) for ethyl acetate extracts, and 1.0 mm and 5.0 mm against *M. luteus* and *B. subtilis* from the butanolic extract. For all assays, ampicillin was used as the control.

The species *T. elliptica* and *T. minuta* were evaluated by Ramirez et al. [201] against bacteria causing periodontal diseases. The MIC of the *T. elliptica* ethanolic extract obtained against *Lactobacillus acidophilus* was 125 mg/mL. This extract had the highest inhibition halo of 13.83 mm against *L. acidophilus* and 14.23 mm against *Porphyromonas gingivalis*, both at the concentration of 500 mg/mL. The *T. minuta* ethanolic extract showed a mean inhibition halo of 16.0 mm (500 mg/mL) only against *P. gingivalis*. Using the *T. minuta* EO, Shirazi et al. [42] evaluated its antibacterial activity against Gram-negative and Gram-positive bacteria, with MICs found against *S. typhi*, *E. coli*, *S. aureus*, and *B. subtilis* of 150 μg/mL, 165 μg/mL, 67 μg/mL, and 75 μg/mL, respectively.

## 11. Antifungal Activity of Plants from the *Tagetes* Genus

The benefit of antimicrobial activity is one of the most proven targets by several authors through studies on the antifungal capacity of plants from this genus [202].

Ayub et al. [199] carried out an experiment to verify the antifungal potential of the *T. erecta* and *T. patula* species. The petals were collected, dried, and ground for the preparation of hexane and methanolic extracts. Spores of the species *Ganoderma lucidum* and *Alternaria alternata* were used. The disc diffusion method was used, with the drug flumequine (30 μg/disc) used as a control. MIC was determined by microdilution with flumequine (1.0 mg/mL) as the control. The results of the test showed that the values for the inhibition zones varied from6.4–8.5 mm and in the control from 20.6–21.8 mm. The MIC was established between 7.5 and 13.3 mg/mL, while it was 0.21–0.30 mg/mL in the control. The *T. patula* species showed higher antifungal activity compared to *T. erecta*, with inhibition zones ranging from 6.5 to 7.3 mm and MIC values from 0.19 to 24 mg/mL.

Romagnoli et al. [203], extracted the EO from dried *T. patula* flowers and investigated its antifungal effect on *Penicillium digitatum* and *Botrytis cinerea* strains. The EO showed remarkable activity in both fungi, reaching 100% inhibition, even at the lowest concentrations. The MIC of the EO against *P. digitatum* presented a low value (1.25 μL/mL), with no colonies being observed at this concentration. *B. cinerea* showed dose-dependent growth inhibition with an MIC of 10 μL/mL. Still with the same species, *T. patula*, Sesan et al. [204] using the hydroalcoholic extract at concentrations of 10% and 5%, analyzed its action on the isolated *Trichoderma viride* (TV 82) strain, a fungal biocontrol agent that can cause infections in humans. The extract was made from fresh biomass such as stems, leaves, flowers, shoots, and bulbs. The *T. patula* extract inhibited the development of the fungus, even when applied at low concentrations (10% and 5%), with inhibition varying between 50 and 54%.

Thembo et al. [162] used the aerial parts from *T. minuta* against isolates from four fungi species of agricultural and clinical importance: *Fusarium verticillioides*, *F. proliferatum*, *Aspergillus flavus*, and *A. parasiticus*. The extraction solvents used were hexane, dichloromethane, methanol, and water. The concentration of the extracts was 10 mg/mL. The drug amphotericin B and the agricultural fungicide *Cantus* were used as positive controls. The MIC was determined by microdilution and some isolates from *F. verticillioides* and *F. proliferatum* strains were sensitive to hexane (0.02–2.5 mg/mL), dichloromethane (0.02–0.32 mg/mL), and methanol (0.02–2.5 mg/mL) extracts with fungistatic action. The aqueous extract had no activity on the fungal strains.

The *Tagetes pusilla* EO was investigated against the *C. albicans* strain using the cavity diffusion method. Alzamora et al. [205] used micoral (100 mg), sporostantin (330 mg), mycostatin (10,000 UI/mL), and oxonasol (200 mg) as controls. The analysis resulted in an inhibition halo greater than 20 mm in diameter, suggesting that the *C. albicans* strain was extremely sensitive to the EO. Ali et al. [90], using the same methodology, tested the EO from *T. minuta* leaves against *C. albicans*. The EO showed good anticandidal activity with a 26-mm inhibition zone, compared to nystatin.

Dutta et al. [206] tested the EO from the *T. patula* leaf on a *C. albicans* strain by disc diffusion. Miconazole (1000 μg/mL) and clotrimazole (1000 μg/mL) were used as controls. The MIC was determined by microdilution with the EO dissolved in 5% dimethyl sulfoxide (DMSO) and then diluted in Sabouraud dextrose broth. The result recorded an inhibition zone of 7.7 mm, a result similar to the inhibition determined for miconazole (10 mm) and clotrimazole (9.3 mm). The MIC for the EO was 3180 μg/mL. *A. niger* and *C. albicans* strains were used by Shirazi et al. [42] to perform experiments with the *T. minuta* EO. MIC was determined, and ketoconazole (10 μg/mL) was used as a control. The MICs for *A. niger* and *C. albicans* were 135 and 115 μg/mL, respectively.

Politi et al. [41] used 70% (5 mg/mL) ethanolic extracts from the aerial parts (stems, leaves, and flowers) of *T. patula* to evaluate the antifungal potential of clinically important fungi by microdilution using amphotericin B (16 mg/mL) as a positive control. Extracts from the aerial parts with flowers, without flowers, and with flower extracts were evaluated. The best results verified against *T. rubrum* were recorded using the extract from aerial parts with flowers (254 μg/mL), whilst against *T. mentagrophytes*, the ethanolic extract from aerial parts with and without flowers were more effective, with MIC values of 573 μg/mL and 625 μg/mL, respectively. Additionally, for *M. canis*, the flower extract (195 μg/mL) stood out, also displaying the lowest inhibitory concentration of all the extracts tested. *Metarhizium anisoplia* and *B. bassiana* were not sensitive to the extracts, which is a good result, considering that these entomopathogenic microorganisms are important for biological control, despite the latter being responsible for hyalohyphomycoses in humans.

Tests with the methanolic extract from the *T. patula* plant against the fungi *B. cinerea*, *Fusarium moniliforme*, and *Pythium ultimum* were performed by Mares and coworkers [207]. The extract was tested in solid medium at concentrations of 5, 10, and 50 μg/mL. Treatments were performed with sunlight (Biolux lamps), ultraviolet (UV)-A, and in the dark with scanning electron microscope readings. For *B. cinerea* under solar irradiation, the inhibition was dose-dependent reaching 39.3% growth in colonies treated with the maximum dose (50 μg/mL). At the same dose, irradiation with UV-A improved the action of the extract with 57.4% inhibition, and, in the dark, a value of 24.8% was observed. For *F. moniliforme* under sunlight, the inhibition observed at 50 mg/mL had the value of 50.9%, while it was 47.3% for UV-A and 33.8% in the dark, while the results for *P. ultimum* were 72.6% (solar), 62.7% (UV-A), and 51.4% (dark).

## 12. Ethnobiology of *Tagetes* Antimicrobial Activity

The scientific literature discussed here revealed the antimicrobial potential of species from the *Tagetes* genus. Four species presented antifungal effects and five presented antibacterial effects. In this sense, species of the *Tagetes* genus had an effect on 17 different fungal strains and 15 different bacterial strains which cause infections in humans. From ethnobiological reports, the *T. filifolia* and *T. lucida* species, indicated for the treatment of diseases related to fungi and bacteria, are yet to be evaluated by the scientific community regarding their antifungal and antibacterial potentials. None of the species which had antimicrobial bioactivity were evaluated in combination with commercial antifungals or antibacterials, where a synergistic effect is often seen as a positive interaction from this association. This investigation was also not mentioned in the methodology and, consequently, results from this ethnobiological research are lacking, which is of the utmost importance, since many people use teas and medications concomitantly.

## 13. Conclusions and Future Perspectives

The *Tagetes* genus is rich in aromatic compounds and resinous exudate, and the EOs of these plants are rich in ocimenes, limonene, terpinene, myrcene, tagetones, dihydrotagetone, and tagetenones, which are the primary odorants, and lower amounts of sesquiterpene hydrocarbons and oxygenated compounds. However, the chemical EO composition is high dependent on several endogenous and exogenous factors, including genetic traits, plant organs (leaves, stems, capitula, or roots), growing, drying, and storage conditions, and stress factors such as adverse climatology and diseases affecting the plant. Moreover, one crucial aspect of the EO composition is the type of extraction, the solvents utilized, and the standardization of the extracts. Thus, plant extracts from different parts of these plants may show different biological abilities, and therefore, can be used in a variety of industries, including cosmetic, pharmaceutical, or food production, due to the presence of biologically active compounds such as 5,7,4′-trimethoxyflavone (apigenin trimethyl ether), patuletin (*O*-methylatedflavanol), quercetagetin and its 7-arabinosyl-galactoside, and other flavonoids, carotenoids (lutein), and thiophene derivatives (α-terthienyl).

A clear-cut and feasible correlation of the biological effects reported for the various EOs and other extracts with their chemical composition is not evident, but some associations can be made. For example, the antimicrobial activity of limonene for medical purposes is well known [208], and its therapeutic effects involving anti-inflammatory, antioxidant, antinoceptive, anticancer, antidiabetic, and antihyperalgesic effects, among others, were extensively studied [209]. In an in vitro study [210], antibacterial and antifungal activities of α-pinene and β-pinene enantiomers were evaluated, showing that positive enantiomers had antimicrobial activity against *C. albicans*, *C. neoformans*, *Rhizopus oryzae*, and methicillin-resistant *S. aureus* (MRSA), while also showing that pinene is useful in formulating strategies to limit *C. albicans* biofilm formation. Marchese et al. [211] proposed a mechanistic viewpoint of antimicrobial activity, against both planktonic and sessile cells belonging to food-decaying microorganisms and human pathogens, of eugenol and EOs containing eugenol. Eugenol interferes with membrane functions or suppresses virulence factors, including toxins, enzymes, and the formation of bacterial and fungal biofilms. Interestingly, the authors suggest a synergist effect of eugenol and other compounds present in EOs such as thymol, carvacrol, and menthol (compounds also present in *Tagetes* EOs). Some studies were conducted with major and common compounds present in the extracts; however, much research is missing with the minor and uncommon compounds, with a lack of investigation into the mechanisms of action. However, in this review, we evidenced the antimicrobial activity of *Tagetes* spp. extracts.

Antioxidant components are commonly used as food preservative applications. In the agriculture field, these plants have high potential uses because their bioactive compounds are involved in defense against parasites (bacteria, fungi, and some insects) and the attraction of pollinators. The *Tagetes* genus is commonly cultivated as an ornamental plant around the world. On the other hand, this plant is used in folk medicine, especially in countries where this plant is native. Today, this plant is used in folk medicine in several countries against signs and symptoms related to bacterial and fungal infection. However, further clinical studies are needed to corroborate these effects in humans.

Among the bioactivities reported for these plants, their properties are interesting to be exploited in the industry. In addition, the use of *Tagetes* products in foods seems to be promising as natural antioxidant agents and antimicrobial preservatives, particularly as novel components of active packaging systems. Finally, extraction methods should be improved to obtain compounds or optimized EO compositions, using food-grade solvents and green extraction methods, e.g., supercritical fluid extraction using CO_2_ or pressurized water extraction.

## Figures and Tables

**Table 1:** Major essential oil (EO) components, as percentages, of *Tagetes patula* aerial parts.

Molecules	India	Egypt	South Africa
(*E*)-β-ocimene (**5**)	16.6–35.3	31.0–43.3	40.4–69.8
dihydrotagetone (**6**)	11.9–48.1	3.0–22.0	5.3–17.7
(*Z*)-tagetone (**7**)	18.6–27.2	4.8–10.7	1.3–12.4
(*Z*)-tagetenone (**8**)	8.1–32.5	4.8–10.3	6.9–21.6
(*E*)-tagetenone (**8**)		4.2–7.8	0.4–9.0
(*E*)-tagetone (**7**)	2.5–6.1	0.6–2.0	0.4–2.4
limonene (**1**)	-	2.9–6.8	tr–9.5
β-myrcene	-	-	tr–1.4
germacrene B	-	1.0–1.3	-
β-caryophyllene (**9**)	-	0.6–1.1	-

tr: traces.

**Table 2:** Major EO components, as percentages, of *T. patula* capitula.

Molecules	India	Venezuela
β-caryophyllene (**9**)	19	23.7
terpinolene (**4**)	7	15.6
(*E*)-β-ocimene (**5**)	12	15.5
δ-elemene	17 *	-
(*Z*)-tagetenone (**8**)	6	-
1,8-cineole	4 *	-
piperitenone	3	-
(*E*)-tagetenone (**8**)	3	-
(*E*)-β-ionone	3	-
alloocimene	2	-
(*Z*)-sabinol	2	-
(*E*)-β–ocimene (**5**)	1.8	-
bicyclogermacrene	1.3	-

* Particularly high content.

**Table 3:** Comparison of the EO components from various parts of *T. erecta*.

Molecules	Aerial Parts	Capitula	Leaves
limonene (**1**)	X	X	X
(*E*)-β-farnesene			X
(*E*)-β-ocimene (**5**)	X	X	
(*Z*)-myroxide		X	
(*Z*)-β-ocimene	X	X	X
1,8-cineole	X		
2-hexen-1-al		X	
aromadendrene		X	
camphene			X
carvacrol	X		X
cyperene			X
d-carvone		X	
dihydrotagetone (**6**)	X		
dipentene	X		X
eudesmol		X	
eugenol (**10**)		X	
geraniol	X		X
geranyl acetate			X
indole	X		X
linalol	X	X	X
linalyl acetate	X	X	X
menthol	X		X
myrcene		X	X
*n*-nonyl aldehyde	X	X	X
nerolidol	X		X
*p*-cymen-9-ol			X
*p*-cymene		X	
*p*-mentha-1,3,8-triene			X
phenylacetaldehyde		X	
phenylethyl alcohol		X	
piperitenone	X	X	X
piperitenone oxide		X	
piperitone	X	X	X
sabinene			X
salicylaldehyde		X	
tagetenones	X		
tagetones	X	X	X
terpinen-4-ol			X
terpinolene (**4**)	X	X	X
thymol	X		X
α-pinene (**2**)	X		X
β-caryophyllene (**9**)	X	X	X
β-elemene			X
β-phellandrene	X		X
β-pinene (**3**)	X		X
γ-elemene			X
γ-muurolene			X
γ-terpinene			X

X means that the chemical compound was detected in the EOs obtained from that specific part of the plant.

**Table 4:** Major EO components, as percentages, of *T. minuta* aerial parts.

Molecules	India	South Africa	Egypt
(*E*)-β-ocimene (**5**)	16.6–35.3	40.4–69.8	31.0–43.3
dihydrotagetone (**6**)	11.9–48.1	5.3–17.7	3.0–22.0
(*Z*)-tagetone (**7**)	18.6–27.2	1.3–12.4	4.8–10.7
(*Z*)-tagetenone (**8**)	8.1–32.5	6.9–21.6	4.8–10.3
(*E*)-tagetenone (**8**)		0.4–9.0	4.2–7.8
(*E*)-tagetone (**7**)	2.5–6.1	0.4–2.4	0.6–2.0
limonene (**1**)	-	tr–9.5	2.9–6.8
β-myrcene	-	tr–1.4	-
germacrene B	-	-	1–1.3
β-caryophyllene (**9**)	-	-	0.6–1.1

tr: traces.

**Table 5:** EOs dominated by (*Z*)-β-ocimene or dihydrotagetone in *T. minuta* aerial parts.

Origin	Chemical Features
EOs from Brazil, France, and Hungary	(*Z*)-β-ocimene > (*Z*)- and (*E*)-tagetenone > (*Z*)- and (*E*)-tagetone and dihydrotagetone
EOs from North America	(*Z*)-β-ocimene > (*Z*)- and (*E*)-tagetenone > dihydrotagetone > (*Z*)- and (*E*)-tagetone
EOs from Rwanda	(*Z*)-β-ocimene > (*Z*)- and (*E*)-tagetone > (*Z*)- and (*E*)-tagetenones > dihydrotagetone
EOs from Kashmir and Himachal Pradesh (India)	(*Z*)- β-ocimene > (*Z*)- and (*E*)-tagetenone > dihydrotagetone > (*Z*)- and (*E*)-tagetone
EOs from Bhutan	(*Z*)-β-ocimene > dihydrotagetone > (*Z*)- and (*E*)-tagetone and tagetenone
EOs from Zambia and Andhra Pradesh (India)	dihydrotagetone > (*Z*)-β-ocimene > (*Z*)- and (*E*)-tagetone > (*Z*)- and (*E*)-tagetenone
EOs from Turkey	dihydrotagetone > (*Z*)-β-ocimene > (*Z*)- and (*E*)-tagetenone > (*Z*)- and (*E*)-tagetone
EOs from Lucknow (India)	dihydrotagetone > (*Z*)- and (*E*)- tagetone > (*Z*)- and (*E*)-tagetenone and (*Z*)-β-ocimene

**Table 6:** Major EO components from *T. minuta* aerial parts.

Molecules	Babu and Kaul 2007 *	Reddy et al. 2016
limonene (**1**)	6.0	1.9
(*E*)-β-ocimene (**5**)	49.3	37.9
dihydrotagetone (**6**)	12.1	12.7
(*E*)-tagetone (**7**)	0.4	1.4
(*Z*)-tagetone (**7**)	3.7	11.8
(*E*)-tagetenone (**8**)	3.0	11.4
(*Z*)-tagetenone (**8**)	3.7	5.4
bicyclogermacrene	-	0.9

* EO obtained by vacuum distillation.

**Table 7:** Major EO components of *T. minuta* aerial parts, harvested at various growth stages.

Molecules	Vegetative Stage	Budding Stage	Full Flower Stage	Seed Stage
(*E*)-β-ocimene (**5**)	3.2	16.6	14.4	23.5
dihydrotagetone (**6**)	54.3	21.9	30.3	29.0
(*E*)-tagetone (**7**)	0.8	3.8	3.4	2.4
(*Z*)-tagetone (**7**)	1.9	23.9	13.7	13.5
(*Z*)-tagetenone (**8**)	0.9	9.9	7.0	5.3
(*E*)-tagetenone (**8**)	0.5	4.2	2.0	6.8

**Table 8:** Major EO components, reported as percentages, of *T. minuta* aerial parts from Kenya.

Molecules	Wanzala et al. 2014 [77]	Kimutai et al. 2017 [78]
(*Z*)-β-ocimene (**5**)	43.8	9.8
dihydrotagetone (**6**)	16.7	21.2
piperitenone	10.2	
(*E*)-tagetone (**7**)	8.7	16.2
3,9-epoxy-p-mentha-1,8(10)diene	6.5	-
(*E*)-β-ocimene (**5**)	3.3	-
(*Z*)-tagetone (**7**)	1.9	14.9
limonene (**1**)	-	7.4
alloocimene	-	6.7
(*Z*)-tagetenone (b)	-	4.1

**Table 9:** List of EO components of *T. minuta* from capitula.

δ-cadinene	acetaldehyde	methyleugenol	(*Z*,*Z*)-alloocimene
**(*E*)-tagetenone (1.8–30.6%)**	acetone	nerolidol	α-cadinol
**(*E*)-tagetone (0.5–3.4%)**	aromadendrene	octanal	α-humulene
(*E*)-α-bergamotene	camphene	octanol	α-p-dimethylstyrene
(*E*)-β-ocimene	carvacrol	*p*-menth-4-en-3-one	α-phellandrene
**(*Z*)-tagetenone (tr–32%)**	decenal	phenylethylalcohol	α-terpinene
**(*Z*)-tagetone (1.8–46%)**	**dihydrotagetone (0.8–15.4%)**	piperitenone	α-thujene
**(*Z*)-β-ocimene (25–47%)**	(*E*)-β-farnesene	piperitone	α-thujone
*(Z)*-β-ocimene epoxide	eugenol	propyl butyrate	β-caryophyllene
2-isobutyl-norbornane	geraniol	**sabinene (0.1–0.6%)**	β-myrcene
2-methylbutyl acetate	isobornyl acetate	salicylaldehyde	β-phellandrene
2-methylethyl butyrate	isopiperitenone	terpinen-4-olo	β-pinene
2-methylethyl propionate	l-carvone	terpinolene	β-thujone
2,3,5-trimethyl furan	**limonene (1.3–3.6%)**	thymol	β-elemene
3-methylbutyl acetate	menthol	thymolhydroquinone dimethyl-ether	
4-methyl-2-pentanone	methyl carvacrol	toluene	
5-isobutyl-3-methyl-2-furancarbaldehyde	methyl chavicol	(*Z*)- and (*E*)-alloocimene	

The main components are reported in bold with their corresponding percentages; tr: traces.

**Table 10:** Major EO components, reported as percentages, of *T. minuta* capitula from Argentina.

Molecules	Gila et al. 2000 [81]	Chamorro et al. 2008 [82]
(*E*)-β-ocimene (**5**)	63.0	28.4–55.3
(*Z*)-β-ocimene (**5**)	13.0–38.0	
(*E*)-tagetenone (**8**)	6.0–16.0	19.0–47.5
(*Z*)-tagetenone (**8**)	0.9–10.18	
α-phellandrene	3.9	-
dihydrotagetone (**6**)	2.0–2.7	3.9–14.3
*o*-cymene	1.74	-
tagetones	-	3.1–14.4
limonene (**1**)	2.1–12.7	4.6–11.1
β-phellandrene	-	0.5–2.5

**Table 11:** Variation in EO components, reported as percentages, of *T. minuta* capitula relative to various agronomical parameters.

Molecules	Kumar et al. 2012 [87]	Kumar et al. 2014 [88]
(*Z*)-β-ocimene (**5**)	21.1–36.5	24.3–25.2
dihydrotagetone (**6**)	1.9–3.9	9.5–9.6
(*Z*)-tagetone (**7**)	0.6–1.9	1.0–1.1
(*E*)-tagetone (**7**)	6.4–14.9	15.0–15.9
(*Z*)-tagetenone (**8**)	4.2–7.8	5.0–5.4
(*E*)-tagetenone (**8**)	28.5–37.1	28.7–30.5
limonene (**1**)	-	4.9

**Table 12:** EO composition in *T. minuta* leaves.

Origin	Chemical Features
EOs from Rwanda	dihydrotagetone > (*Z*)-tagetone > (*Z*)-tagetenone > (*E*)-tagetone > (*Z*)-β-ocimene > (*E*)-tagetenone
EOs from Mukoni (Rwanda)	(*E*)-tagetenone > (*Z*)-tagetone > dihydrotagetone, (*E*)-tagetone and (*Z*)-β-ocimene
EOs from Uttar Pradesh (India)	(*Z*)-tagetone > (*Z*)-tagetenone > dihydrotagetone, (*E*)-tagetone and (*Z*)-β-ocimene > (*E*)-tagetone
EOs from France	(*Z*)-tagetenone > (*Z*)-β-ocimene > (*Z*)-tagetone > dihydrotagetone

**Table 13:** EO composition, reported as percentages, of *T. minuta* leaves upon variation of agronomical parameters.

Molecules	Kumar et al. 2012 [87]	Kumar et al. 2014 [88]
(*Z*)-β-ocimene (**5**)	5.7–11.5	10.5–10.7
dihydrotagetone (**6**)	24.6–39.1	43.7–45.8
(*Z*)-tagetone (**7**)	1.7–2.8	1.1–1.4
(*E*)-tagetone (**7**)	28.1–34.5	19.7–21.5
(*Z*)-tagetenone (**8**)	1.4–3.1	1.2
(*E*)-tagetenone (**8**)	9.6–18.1	6.8–7.2
limonene (**1**)	-	6.9–7.1

**Table 14:** *Tagetes* spp. activity against microorganisms in vitro.

Plant Species	Microbial Strain	References
*T. erecta*	*Escherichia coli*, *Bacillus subtilis*, *Klebsiella pneumoniae*, *Pseudomonas aeruginosa*, *Staphylococcus aureus*, *C. albicans*, and *S. cerevisiae*	[163]
	*Alcaligenes faecalis*, *Bacillus cereus*, *Campylobacter coli*, *E. coli*, *K. pneumoniae*, *P. aeruginosa*, *Proteus vulgaris*, *Streptococcus mutans*, and *Streptococcus pyogenes*	[164]
	*B. cereus*, *B. subtilis*, *S. aureus*, *Staphylococcus albus*, *Bacillus megaterium*, *Listeria monocytogenes*, *Corynebacterium rubrum*, *E. coli*, *Pseudomonas pseudoalcaligenes*, *Pseudomonas testosterone*, *Proteus morganii*, *P. aeruginosa*, *Enterobacter aerogenes*, *K. pneumoniae*, *Proteus mirabilis*, *C. albicans*, *Cryptococcus neoformans*, *Candida glabrata*, and *Candida apicola*	[35]
	*E. coli*	[165]
	*C. albicans*	[166]
*T. erecta* and *T. patula*	*E. coli*, *P. vulgaris*, *P. mirabilis*, *Aeromonas sobria*, *Aeromonas hydrophila*, *Plesiomonas shigelloides*, *Salmonella enterica* ser. Typhi, *Salmonella enterica* ser. Typhimurium, *Salmonella enterica* ser. Aboni, *Salmonella enterica* ser. Enteritidis, *B. subtilis*, *B. cereus*, *Bacillus circulans*, and *S. aureus*	[167]
*T. patula*	*Sarcina lutea*, *B. megaterium*, *E. coli*, and *Vibrio parahaemolyticus*	[168]
	*Corynebacterium* spp., *Staphylococcus* spp., *Streptococcus* spp., and *Micrococcus luteus*	[169]
*T. lucida*	*E. coli*, *Aeromonas hominis*, *P. aeruginosa*, *Enterobacter alcalifaciens*, and *E. coli*	[170]
	*E. coli*, *P. mirabilis*, *K. pneumoniae*, and *Salmonella* spp.	[160]
	*Shigella boydii*, *S. aureus*, *Staphylococcus epidermidis*, *P. aeruginosa*, *B. subtilis*, *S. lutea*, and *Vibrio cholerae*	[159]
*T. minuta*	*Salmonella typhi*, *E. coli*, *S. aureus*, *B. subtilis*, *A. niger*, and *C. albicans*	[42]
	*B. cereus*, *B. subtilis*, *S. aureus*, *Streptococcus faecalis*, *E. coli*, *P. mirabilis*, *P. aeruginosa*, and *S. typhi*	[171]
*T. terniflora*	*E. coli*, *S. aureus*, *S. epidermidis*, *P. aeruginosa*, *M. luteus*, *Z. mobilis*, *L. plantarum*, and *S. cerevisiae*	[158]

**Table 15:** Use of *Tagetes* genus in folk medicine against signs and symptoms related to bacterial and fungal infection.

Specie	Use	Used Part	Preparations	Utilization Method	References	Country
*T. erecta*	Gastrointestinal disorders, diarrhea, stomachache, dysentery, ulcer, dental problems, skin diseases, rash, cut, wounds, boils, sore throat, cough	Flowers, leaves	Infusion, crushed leaves, juice from the leaves, paste of leaves, decoction	Oral/local application for wounds and dental problems; oral as leaf juice; local application: paste of leaves used in the treatment of ulcers and wounds; topical: leaves boiled in water to wash affected area and to relieve itchiness and rash	[130,187,188,189,190,191,192]	Mexico, India, Belize, Bangladesh
*T. filifolia*	Severe colic, diarrhea, stomachache	Whole plant, fresh or dried	Not informed	Oral: 10 g per L mixed with Poleo, Manzanilla, Muña, or Chancas de comida and Hinojo; 3 cups daily for 1 week to 1 month	[193,194]	Peru, Mexico
*T. lucida*	Digestive problems, gum diseases, caries, toothache, rheumatism, ulcers in mucus membranes and vaginal fluids, antiseptic, bronchitis	Aerial parts	Infusion, decoction	Topical, mouthwash, local application	[124,195]	Mexico
*T. minuta*	diarrhea, digestive for children, wounds in the mouth	Leaves, seeds	Not informed	Topical	[113,140,196]	Ethiopia, Pakistan, Argentina

## References

[1] Maciel M.A.M., Pinto A.C., Veiga V.F., Grynberg N.F., Echevarria A. (2002). Medicinal plants: The need for multidisciplinary scientific studies. Quím. Nova.

[2] Sharifi-Rad J., Fallah F., Setzer W., Entezari R.H., Sharifi-Rad M. (2016). *Tordylium persicum* boiss. & hausskn extract: A possible alternative for treatment of pediatric infectious diseases. Cell. Mol. Biol..

[3] Mishra A.P., Saklani S., Sharifi-Rad M., Iriti M., Salehi B., Maurya V.K., Rauf A., Milella L., Rajabi S., Baghalpour N. (2018). Antibacterial potential of *Saussurea obvallata* petroleum ether extract: A spiritually revered medicinal plant. Cell. Mol. Biol..

[4] Sharifi-Rad J., Sharifi-Rad M., Salehi B., Iriti M., Roointan A., Mnayer D., Soltani-Nejad A., Afshari A. (2018). In vitro and in vivo assessment of free radical scavenging and antioxidant activities of *Veronica persica* poir. Cell. Mol. Biol..

[5] Sharifi-Rad M., Salehi B., Sharifi-Rad J., Setzer W.N., Iriti M. (2018). *Pulicaria vulgaris* gaertn. Essential oil: An alternative or complementary treatment for leishmaniasis. Cell. Mol. Biol..

[6] Sharifi-Rad J., Salehi B., Stojanović-Radić Z., Fokou P., Sharifi-Rad M., Mahady G., Masjedi M., Lawal T., Ayatollahi S., Masjedi J. (2017). Medicinal plants used in the treatment of tuberculosis—Ethnobotanical and ethnopharmacological approaches. Biotechnol. Adv..

[7] Salehi B., Ayatollahi S.A., Segura-Carretero A., Kobarfard F., Contreras M., Faizi M., Sharifi-Rad M., Tabatabai S.A., Sharifi-Rad J. (2017). Bioactive chemical compounds in *Eremurus persicus* (joub. & spach) boiss. Essential oil and their health implications. Cell. Mol. Biol..

[8] Mishra A., Saklani S., Salehi B., Parcha V., Sharifi-Rad M., Milella L., Iriti M., Sharifi-Rad J., Srivastava M. (2018). *Satyrium nepalense*, a high altitude medicinal orchid of indian himalayan region: Chemical profile and biological activities of tuber extracts. Cell. Mol. Biol..

[9] Sharifi-Rad J., Tayeboon G.S., Niknam F., Sharifi-Rad M., Mohajeri M., Salehi B., Iriti M., Sharifi-Rad M. (2018). *Veronica persica* Poir. Extract–antibacterial, antifungal and scolicidal activities, and inhibitory potential on acetylcholinesterase, tyrosinase, lipoxygenase and xanthine oxidase. Cell. Mol. Biol..

[10] Sharifi-Rad M., Nazaruk J., Polito L., Morais-Braga M.F.B., Rocha J.E., Coutinho H.D.M., Salehi B., Tabanelli G., Montanari C., del Mar Contreras M. (2018). *Matricaria* genus as a source of antimicrobial agents: From farm to pharmacy and food applications. Microbiol. Res..

[11] Sharifi-Rad M., Varoni E.M., Salehi B., Sharifi-Rad J., Matthews K.R., Ayatollahi S.A., Kobarfard F., Ibrahim S.A., Mnayer D., Zakaria Z.A. (2017). Plants of the genus *Zingiber* as a source of bioactive phytochemicals: From tradition to pharmacy. Molecules.

[12] Sharifi-Rad J., Salehi B., Varoni E.M., Sharopov F., Yousaf Z., Ayatollahi S.A., Kobarfard F., Sharifi-Rad M., Afdjei M.H., Sharifi-Rad M. (2017). Plants of the *Melaleuca* genus as antimicrobial agents: From farm to pharmacy. Phytother. Res..

[13] Boutaoui N., Zaiter L., Benayache F., Benayache S., Cacciagrano F., Cesa S., Secci D., Carradori S., Giusti A.M., Campestre C. (2018). *Atriplex mollis* Desf. aerial parts: Extraction procedures, secondary metabolites and color analysis. Molecules.

[14] Sharifi-Rad M., Varoni E.M., Iriti M., Martorell M., Setzer W.N., Contreras M., Salehi B., Soltani-Nejad A., Rajabi S., Tajbakhsh M. (2018). Carvacrol and human health: A comprehensive review. Phytother. Res..

[15] Bagheri G., Mirzaei M., Mehrabi R., Sharifi-Rad J. (2016). Cytotoxic and antioxidant activities of *Alstonia scholaris, Alstonia venenata* and *Moringa oleifera* plants from India. Jundishapur J. Nat. Pharm. Prod..

[16] Stojanović-Radić Z., Pejčić M., Stojanović N., Sharifi-Rad J., Stanković N. (2016). Potential of *Ocimum basilicum* L. And *Salvia officinalis* L. Essential oils against biofilms of *P. aeruginosa* clinical isolates. Cell. Mol. Biol..

[17] Salehi B., Sharopov F., Martorell M., Rajkovic J., Ademiluyi A., Sharifi-Rad M., Fokou P., Martins N., Iriti M., Sharifi-Rad J. (2018). Phytochemicals in *Helicobacter pylori* infections: What are we doing now?. Int. J. Mol. Sci..

[18] Abdolshahi A., Naybandi-Atashi S., Heydari-Majd M., Salehi B., Kobarfard F., Ayatollahi S., Ata A., Tabanelli G., Sharifi-Rad M., Montanari C. (2018). Antibacterial activity of some Lamiaceae species against *Staphylococcus aureus* in yoghurt-based drink (doogh). Cell. Mol. Biol..

[19] Sharifi-Rad J., Iriti M., Setzer W.N., Sharifi-Rad M., Roointan A., Salehi B. (2018). Antiviral activity of *Veronica persica* Poir. on herpes virus infection. Cell. Mol. Biol..

[20] Devika R., Koilpillai J. (2015). Anti-inflammatory effect of bioactive compounds of *Tagetes erecta* (Linn.) flower extract. J. Pure Appl. Microbiol..

[21] Sharifi-Rad J., Salehi B., Schnitzler P., Ayatollahi S., Kobarfard F., Fathi M., Eisazadeh M., Sharifi-Rad M. (2017). Susceptibility of herpes simplex virus type 1 to monoterpenes thymol, carvacrol, p-cymene and essential oils of *Sinapis arvensis* L., *Lallemantia royleana* Benth. and *Pulicaria vulgaris* Gaertn. Cell. Mol. Biol..

[22] Salehi B., Zucca P., Sharifi-Rad M., Pezzani R., Rajabi S., Setzer W.N., Varoni E.M., Iriti M., Kobarfard F., Sharifi-Rad J. (2018). Phytotherapeutics in cancer invasion and metastasis. Phytother. Res..

[23] Salehi B., Mishra A.P., Shukla I., Sharifi-Rad M., del Mar Contreras M., Segura-Carretero A., Fathi H., Nasri Nasrabadi N., Kobarfard F., Sharifi-Rad J. (2018). Thymol, thyme and other plant sources: Health and potential uses. Phytother. Res..

[24] Snow Setzer M., Sharifi-Rad J., Setzer W.N. (2016). The search for herbal antibiotics: An in-silico investigation of antibacterial phytochemicals. Antibiotics.

[25] Sharifi-Rad M., Mnayer D., Flaviana Bezerra Morais-Braga M., Nályda Pereira Carneiro J., Fonseca Bezerra C., Douglas Melo Coutinho H., Salehi B., Martorell M., del Mar Contreras M., Soltani-Nejad A. (2018). *Echinacea* plants as antioxidant and antibacterial agents: From traditional medicine to biotechnological applications. Phytother. Res..

[26] Sharifi-Rad M., Fokou P., Sharopov F., Martorell M., Ademiluyi A., Rajkovic J., Salehi B., Martins N., Iriti M., Sharifi-Rad J. (2018). Antiulcer agents: From plant extracts to phytochemicals in healing promotion. Molecules.

[27] Prakash A.M., Sharifi-Rad M., Shariati M., Mabkhot Y., Al-Showiman S., Rauf A., Salehi B., Župunski M., Gusain P., Sharifi-Rad J. (2018). Bioactive compounds and health benefits of edible rumex species—A review. Cell. Mol. Biol..

[28] Sharifi-Rad J., Roointan A., Setzer W., Sharifi-Rad M., Iriti M., Salehi B. (2018). Susceptibility of leishmania major to *Veronica persica* Poir. extracts-in vitro and in vivo assays. Cell. Mol. Biol..

[29] Locatelli M., Zengin G., Uysal A., Carradori S., De Luca E., Bellagamba G., Aktumsek A., Lazarova I. (2017). Multicomponent pattern and biological activities of seven *Asphodeline* taxa: Potential sources of natural-functional ingredients for bioactive formulations. J. Enzyme Inhib. Med. Chem..

[30] Soule J. Infrageneric systematics of tagetes. Proceedings of the International Compositae Conference, Compositae: Systematics.

[31] Babu K.G., Kaul V. (2007). Variations in quantitative and qualitative characteristics of wild marigold (*Tagetes minuta* L.) oils distilled under vacuum and at NPT. Ind. Crops Prod..

[32] Politi F.A.S., Souza A.A.J., Fantatto R.R., Pietro R., Barioni W.J., Rabelo M.D., Bizzo H.R., de Souza Chagas A.C., Furlan M. (2017). Chemical composition and in vitro anthelmintic activity of extracts of *Tagetes patula* against a multidrug-resistant isolate of *Haemonchus contortus*. Chem. Biodivers..

[33] Lawrence B. (1985). Essential oils of the *tagetes* genus. Perfum. Flavor.

[34] Kashif M., Bano S., Naqvi S., Faizi S., Lubna, Ahmed Mesaik M., Azeemi K.S., Farooq A.D. (2015). Cytotoxic and antioxidant properties of phenolic compounds from *Tagetes patula* flower. Pharm. Biol..

[35] Padalia H., Chanda S. (2015). Antimicrobial efficacy of different solvent extracts of *Tagetes erecta* L. Flower, alone and in combination with antibiotics. Appl. Microbiol. Open Access.

[36] Politi F.A., Nascimento J.D., da Silva A.A., Moro I.J., Garcia M.L., Guido R.V., Pietro R.C., Godinho A.F., Furlan M. (2017). Insecticidal activity of an essential oil of *Tagetes patula* L. (asteraceae) on common bed bug *Cimex lectularius* L. And molecular docking of major compounds at the catalytic site of clache1. Parasitol. Res..

[37] Girón L.M., Freire V., Alonzo A., Cáceres A. (1991). Ethnobotanical survey of the medicinal flora used by the caribs of guatemala. J. Ethnopharmacol..

[38] Laferriere J.E., Weber C.W., Kohlhepp E.A. (1991). Mineral composition of some traditional mexican teas. Plant. Foods Hum. Nutr. (Former. Qual. Plant.).

[39] Marotti M., Piccaglia R., Biavati B., Marotti I. (2004). Characterization and yield evaluation of essential oils from different *tagetes* species. J. Essent. Oil Res..

[40] Burt S. (2004). Essential oils: Their antibacterial properties and potential applications in foods—A review. Int. J. Food Microbiol..

[41] Politi F.A.S., Queiroz-Fernandes G.M., Rodrigues E.R., Freitas J.A., Pietro R. (2016). Antifungal, antiradical and cytotoxic activities of extractives obtained from *Tagetes patula* L. (asteraceae), a potential acaricide plant species. Microb. Pathog..

[42] Shirazi M.T., Gholami H., Kavoosi G., Rowshan V., Tafsiry A. (2014). Chemical composition, antioxidant, antimicrobial and cytotoxic activities of *Tagetes minuta* and *Ocimum basilicum* essential oils. Food Sci. Nutr..

[43] Lu H., Yang S., Ma H., Han Z., Zhang Y. (2016). Bioassay-guided separation and identification of anticancer compounds in *Tagetes erecta* L. Flowers. Anal. Methods.

[44] Khalil M., Raila J., Ali M., Islam K.M., Schenk R., Krause J.-P., Schweigert F.J., Rawel H. (2012). Stability and bioavailability of lutein ester supplements from *tagetes* flower prepared under food processing conditions. J. Funct. Foods.

[45] Singh V., Singh B., Kaul V.K. (2003). Domestication of wild marigold (*Tagetes minuta* L.) as a potential economic crop in western himalaya and north indian plains. Econ. Bot..

[46] Priyanka D., Shalini T., Navneet V. (2013). A brief study of marigold (*Tagetes species*): A review. Int. Res. J. Pharm..

[47] Thappa R., Agarwal S., Kalia N., Kapoor R. (1993). Changes in chemical composition of *Tagetes minuta* oil at various stages of flowering and fruiting. J. Essent. Oil Res..

[48] Kumar B., Gupta A., Verma A., Dubey A. (2008). Comparative seed germination of *Tagetes minuta*. J. Trop. Med..

[49] Prakasa Rao E.V.S., Puttanna K., Ramesh S. (2000). Effect of nitrogen and harvest stage on the yield and oil quality of *Tagetes minuta* L. In tropical india. J. Herbs Spices Med. Plants.

[50] Singh S., Singh V., Babu G., VK K., Ahuja P. (2006). Techno-economic feasibility of wild marigold (*Tagetes minuta)* oil production in himachal pradesh. J. Non-Timber For. Prod..

[51] Langenheim J.H. (2003). Plant. Resins—Chemistry, Evolution Ecology and Ethnobotany.

[52] The Plant List A Working List of all Plant Species. http://www.theplantlist.org/.

[53] Tropicos Missouri Botanical Garden. http://tropicos.Org/namesearch.Aspx?Name=tagetes&commonname=.

[54] Tisserand R., Young R. (2014). Essential Oil Safety: A Guide for Health Care Professionals.

[55] Gupta P., Vasudeva N. (2012). Marigold: A potential ornamental plant drug. Hamdard Med..

[56] Singh P., Krishna A., Kumar V., Krishna S., Singh K., Gupta M., Singh S. (2015). Chemistry and biology of industrial crop *tagetes* species: A review. J. Essent. Oil Res..

[57] Dharmagadda V.S., Naik S.N., Mittal P.K., Vasudevan P. (2005). Larvicidal activity of *Tagetes patula* essential oil against three mosquito species. Bioresour. Technol..

[58] Politi F.A., de Souza-Moreira T.M., Rodrigues E.R., de Queiroz G.M., Figueira G.M., Januario A.H., Berenger J.M., Socolovschi C., Parola P., Pietro R.C. (2013). Chemical characterization and acaricide potential of essential oil from aerial parts of *Tagetes patula* L. (Asteraceae) against engorged adult females of *Rhipicephalus sanguineus* (latreille, 1806). Parasitol. Res..

[59] Armas K., Rojas J., Rojas L., Morales A. (2012). Comparative study of the chemical composition of essential oils of five *tagetes* species collected in Venezuela. Nat. Prod. Commun..

[60] Prakash O., Rout P.K., Chanotiya C.S., Misra L.N. (2012). Composition of essential oil, concrete, absolute and spme analysis of *Tagetes patula* capitula. Ind. Crops Prod..

[61] Li J., Song S.D., Zhang R.N., Liu N., Li C.C. (2011). Chemical components and nitrite cleaning activity of essential oil from *Tagetes erecta* L. Leaf. Adv. Mater. Res..

[62] Marques M.M., Morais S.M., Vieira I.G., Vieira M.G., Raquel A., Silva A., De Almeida R.R., Guedes M.I. (2011). Larvicidal activity of *Tagetes erecta* against *Aedes aegypti*. J. Am. Mosq. Control Assoc..

[63] Lawrence B.M. (1992). Progress in essential oils: *Tagetes* oil. Perfum. Flavor..

[64] Lawrence B.M. (1996). Progress in essential oils: *Tagetes* oil. Perfum. Flavor..

[65] Lawrence B.M. (2000). Progress in essential oils: *Tagetes* oil. Perfum. Flavor..

[66] Lawrence B.M. (2006). Progress in essential oils: *Tagetes* oil. Perfum. Flavor..

[67] Lawrence B.M. (2009). Progress in essential oils: *Tagetes* oil. Perfum. Flavor..

[68] Burfield T. (2017). Natural Aromatic Materials: Odours & Origins.

[69] Juliani H., Biurrun F., Koroch A., Oliva M., Demo M., Trippi V., Zygadlo J. (2002). Chemical constituents and antimicrobial activity of the essential oil of *Lantana xenica*. Planta Med..

[70] Reddy S.G., Kirti Dolma S., Koundal R., Singh B. (2016). Chemical composition and insecticidal activities of essential oils against diamondback moth, *Plutella xylostella* (L.) (lepidoptera: Yponomeutidae). Nat. Prod. Res..

[71] Ramaroson-Raonizafinimanana B., Ramanoelina P.A.R., Rasoarahona J.R.E., Gaydou E.M. (2009). Chemical compositions of aerial part of *Tagetes minuta* L. Chemotype essential oils from madagascar. J. Essent. Oil Res..

[72] Breme K., Tournayre P., Fernandez X., Meierhenrich U.J., Brevard H., Joulain D., Berdague J.L. (2009). Identification of odor impact compounds of *Tagetes minuta* L. Essential oil: Comparison of two gc-olfactometry methods. J. Agric. Food Chem..

[73] Nchu F., Magano S.R., Eloff J.N. (2012). In vitro anti-tick properties of the essential oil of *Tagetes minuta* L. (Asteraceae) on *Hyalomma rufipes* (acari: Ixodidae). Onderstepoort J. Vet. Res..

[74] Gillij V.G., Gleiser R.M., Zygadlo J.A. (2008). Mesquito repellent activity of essential oils of aromatic plants growing in argentina. Bioresour. Technol..

[75] Garcia M.V., Matias J., Barros J.C., de Lima D.P., Lopes Rda S., Andreotti R. (2012). Chemical identification of *Tagetes minuta* linnaeus (Asteraceae) essential oil and its acaricidal effect on ticks. Rev. Bras. Parasitol. Vet..

[76] Macedo I.T., de Oliveira L.M., Camurca-Vasconcelos A.L., Ribeiro W.L., dos Santos J.M., de Morais S.M., de Paula H.C., Bevilaqua C.M. (2013). In vitro effects of *Coriandrum sativum*, *Tagetes minuta*, *Alpinia zerumbet* and *Lantana camara* essential oils on *Haemonchus contortus*. Rev. Bras. Parasitol. Vet..

[77] Wanzala W., Hassanali A., Mukabana W.R., Takken W. (2014). Repellent activities of essential oils of some plants used traditionally to control the brown ear tick, *Rhipicephalus appendiculatus*. J. Parasitol. Res..

[78] Kimutai A., Ngeiywa M., Mulaa M., Njagi P.G., Ingonga J., Nyamwamu L.B., Ombati C., Ngumbi P. (2017). Repellent effects of the essential oils of *Cymbopogon citratus* and *Tagetes minuta* on the sandfly, phlebotomus duboscqi. BMC Res. Notes.

[79] Gakuubi M.M., Wagacha J.M., Dossaji S.F., Wanzala W. (2016). Chemical composition and antibacterial activity of essential oils of *Tagetes minuta* (Asteraceae) against selected plant pathogenic bacteria. Int. J. Microbiol..

[80] Giarratana F., Muscolino D., Ziino G., Giuffrida A., Marotta S.M., Lo Presti V., Chiofalo V., Panebianco A. (2017). Activity of *Tagetes minuta* linnaeus (Asteraceae) essential oil against l3 anisakis larvae type 1. Asian Pac. J. Trop. Med..

[81] Gila A., Ghersa C.M., Leicach S. (2000). Essential oil yield and composition of *Tagetes minuta* accessions from argentina. Biochem. Syst. Ecol..

[82] Chamorro E.R., Ballerini G., Sequeira A.F., Velasco G.A., Zalazar M.F. (2008). Chemical composition of essential oil from *Tagetes minuta* leaves and flowers. J. Argent. Chem. Soc..

[83] Worku T., Bertoldi M., Franz C., Mathe A., Buchbauer G. (1996). Essential oils at different development stages of ethiopian *Tagetes minuta* L.. Essential Oils: Basic and Applied Research.

[84] Chalchat J., Gary J.C., Muhayimana R.P. (1995). Essential oil of *Tagetes minuta* from rwanda and france: Chemical composition according to harvesting, location, growing stage and plant part. J. Essent. Oil Res..

[85] Lawrence B.M. (1992). Progress in essential oils. Perfum. Flavor..

[86] Moghaddam M., Omidbiagi R., Sefidkon F. (2007). Changes in content and chemical composition of *Tagetes minuta* oil at various harvest times. J. Essent. Oil Res..

[87] Kumar R., Ramesh K., Pathania M.V., Singh B. (2012). Effect of transplanting date on growth, yield and oil quality of *Tagetes minuta* L. In mid hill of north-western Himalaya. J. Essent. Oil Bear. Plants.

[88] Kumar R., Sharma S., Ramesh K., Pathasia V., Prasad R. (2014). Irradiance stress and plant spacing effect on growth, biomass and quality of wild Marigold oil (*Tagetes minuta* L.)—An industrial crop in western Himalaya. J. Essent. Oil Res..

[89] Mohamed M.A., Harris P.J., Henderson J., Senatore F. (2002). Effect of drought stress on the yield and composition of volatile oils of drought-tolerant and non-drought-tolerant clones of *Tagetes minuta*. Planta Med..

[90] Ali N.A., Sharopov F.S., Al-Kaf A.G., Hill G.M., Arnold N., Al-Sokari S.S., Setzer W.N., Wessjohann L. (2014). Composition of essential oil from *Tagetes minuta* and its cytotoxic, antioxidant and antimicrobial activities. Nat. Prod. Commun..

[91] Karimian P., Kavoosi G., Amirghofran Z. (2014). Anti-oxidative and anti-inflammatory effects of *Tagetes minuta* essential oil in activated macrophages. Asian Pac. J. Trop. Biomed..

[92] Ciccio J.F. (2004). A source of almost pure methyl chavicol: Volatile oil from the aerial parts of *Tagetes lucida* (Asteraceae) cultivated in costa rica. Rev. Biol. Trop..

[93] Bicchi C., Fresia M., Rubiolo P., Monti D., Franz C., Goehler I. (1997). Constituents of *Tagetes lucida* cav. Ssp. Lucida essential oil. Flavour Fragr. J..

[94] Goehler I. (2006). Domestikation von Medizinalp Anzen und Untersuchungen Zur Inkulturnahme von Tagetes lucida cav.

[95] Caballero-Gallardo K., Olivero-Verbel J., Stashenko E.E. (2011). Repellent activity of essential oils and some of their individual constituents against *Tribolium castaneum* herbst. J. Agric. Food Chem..

[96] Vera S.S., Zambrano D.F., Mendez-Sanchez S.C., Rodriguez-Sanabria F., Stashenko E.E., Duque Luna J.E. (2014). Essential oils with insecticidal activity against larvae of *Aedes aegypti* (Diptera: Culicidae). Parasitol. Res..

[97] Omer E.A., Hendawy S.F., Ismail R.F., Petretto G.L., Rourke J.P., Pintore G. (2017). Acclimatization study of *Tagetes lucida* L. in Egypt and the chemical characterization of its essential oils. Nat. Prod. Res..

[98] Gleiser R.M., Bonino M.A., Zygadlo J.A. (2011). Repellence of essential oils of aromatic plants growing in Argentina against *Aedes aegypti* (Diptera: Culicidae). Parasitol. Res..

[99] Stefanazzi N., Stadler T., Ferrero A. (2011). Composition and toxic, repellent and feeding deterrent activity of essential oils against the stored-grain pests *Tribolium castaneum* (Coleoptera: Tenebrionidae) and *Sitophilus oryzae* (Coleoptera: Curculionidae). Pest. Manag. Sci..

[100] Chopa C.S., Descamps L.R. (2012). Composition and biological activity of essential oils against *Metopolophium dirhodum* (Hemiptera: Aphididae) cereal crop pest. Pest. Manag. Sci..

[101] Hethelyi E., Tetenyi P., Dabi E., Danos B. (1987). The role of mass spectrometry in medicinal plant research. Biomed. Environ. Mass Spectrom..

[102] Pichette A., Garneau F.-X., Collin G., Jean F.-I., Gagnon H., Lopez Arze J.B. (2005). Essential oils from Bolivia. Iv. Compositae: *Tagetes* aff. *Maxima* kuntze and *Tagetes multiflora* h.B.K. J. Essent. Oil Res..

[103] Tucker A., Maciarello M.J. (1996). Volatile leaf oil of tagetes lemmonii gray. J. Essent. Oil Res..

[104] Lopez S.B., Lopez M.L., Aragon L.M., Tereschuk M.L., Slanis A.C., Feresin G.E., Zygadlo J.A., Tapia A.A. (2011). Composition and anti-insect activity of essential oils from *tagetes* L. Species (Asteraceae, Helenieae) on ceratitis capitata wiedemann and triatoma infestans klug. J. Agric. Food Chem..

[105] Buitrago D., Rojas L.B., Rojas J., Morales A. (2010). Volatile compounds from *Tagetes pusilla* (Asteraceae) collected from the Venezuela andes. Nat. Prod. Commun..

[106] Lima B., Agüero M.B., Zygadlo J.A., Tapia A., Solis C., Rojas de Arias A., Yaluff G., Zacchino S., Feresin G.E., Schmeda-Hirschmann G. (2009). Antimicrobial activity of extracts, essential oil and metabolites obtained from *Tagetes mendocina*. J. Chil. Chem. Soc..

[107] Rahman A. (2013). An ethnobotanical investigation on asteraceae family at Rajshahi, Bangladesh. J. Bus. Admin. Manag. Sci. Res..

[108] Parvaiz M. (2014). Ethnobotanical studies on plant resources of mangowal, district Gujrat, Punjab, Pakistan. Avicenna J. Phytomed..

[109] Moreno-Salazar S.F., Robles-Zepeda R.E., Johnson D.E. (2008). Plant folk medicines for gastrointestinal disorders among the main tribes of Sonora, Mexico. Fitoterapia.

[110] Svetaz L., Zuljan F., Derita M., Petenatti E., Tamayo G., Caceres A., Cechinel V., Gimenez A., Pinzon R., Zacchino S.A. (2010). Value of the ethnomedical information for the discovery of plants with antifungal properties. A survey among seven latin american countries. J. Ethnopharmacol..

[111] Alonso J., Desmarchelier C. (2005). Plantas Medicinales Autóctonas de la Argentina. Bases Científicas Para su Aplicación En Atención Primaria de Salud.

[112] Njoroge G.N., Bussmann R.W. (2007). Ethnotherapeautic management of skin diseases among the kikuyus of central Kenya. J. Ethnopharmacol..

[113] Rahman I.U., Ijaz F., Iqbal Z., Afzal A., Ali N., Afzal M., Khan M.A., Muhammad S., Qadir G., Asif M. (2016). A novel survey of the ethno medicinal knowledge of dental problems in Manoor valley (northern Himalaya), Pakistan. J. Ethnopharmacol..

[114] Ata S., Farooq F., Javed S. (2011). Elemental profile of 24 common medicinal plants of pakistan and its direct link with traditional uses. J. Med. Plants Res..

[115] Kujawska M., Hilgert N.I. (2014). Phytotherapy of polish migrants in misiones, argentina: Legacy and acquired plant species. J. Ethnopharmacol..

[116] Ijaz F., Iqbal Z., Rahman I.U., Alam J., Khan S.M., Shah G.M., Khan K., Afzal A. (2016). Investigation of traditional medicinal floral knowledge of Sarban hills, Abbottabad, kp, Pakistan. J. Ethnopharmacol..

[117] Hamill F.A., Apio S., Mubiru N.K., Bukenya-Ziraba R., Mosango M., Maganyi O.W., Soejarto D.D. (2003). Traditional herbal drugs of southern Uganda, ii: Literature analysis and antimicrobial assays. J. Ethnopharmacol..

[118] Bourdy G., Chāvez de Michel L.R., Roca-Coulthard A. (2004). Pharmacopoeia in a shamanistic society: The izoceño-guaraní (Bolivian chaco). J. Ethnopharmacol..

[119] Gunes S., Savran A., Paksoy M.Y., Kosar M., Cakilcioglu U. (2017). Ethnopharmacological survey of medicinal plants in karaisali and its surrounding (adana-Turkey). J. Herb. Med..

[120] Teixidor-Toneu I., Martin G.J., Ouhammou A., Puri R.K., Hawkins J.A. (2016). An ethnomedicinal survey of a tashelhit-speaking community in the high atlas, Morocco. J. Ethnopharmacol..

[121] Agra M.F., Baracho G.S., Nurit K., Basílio I.J., Coelho V.P. (2007). Medicinal and poisonous diversity of the flora of “cariri paraibano”, Brazil. J. Ethnopharmacol..

[122] Adams M., Gmünder F., Hamburger M. (2007). Plants traditionally used in age related brain disorders—A survey of ethnobotanical literature. J. Ethnopharmacol..

[123] Guadarrama-Cruz G., Alarcon-Aguilar F.J., Lezama-Velasco R., Vazquez-Palacios G., Bonilla-Jaime H. (2008). Antidepressant-like effects of *Tagetes lucida* cav. In the forced swimming test. J. Ethnopharmacol..

[124] Pérez-Ortega G., González-Trujano M.E., Ángeles-López G.E., Brindis F., Vibrans H., Reyes-Chilpa R. (2016). *Tagetes lucida* cav.: Ethnobotany, phytochemistry and pharmacology of its tranquilizing properties. J. Ethnopharmacol..

[125] Gutierrez S.L.G., Chilpa R.R., Jaime H.B. (2014). Medicinal plants for the treatment of “nervios”, anxiety, and depression in Mexican traditional medicine. Rev. Bras. Farmacogn.-Braz. J. Pharmacogn..

[126] Leonti M., Vibrans H., Sticher O., Heinrich M. (2001). Ethnopharmacology of the popoluca, Mexico: An evaluation. J. Pharm. Pharmacol..

[127] García-Hernández K.Y., Vibrans H., Rivas-Guevara M., Aguilar-Contreras A. (2015). This plant treats that illness? The hot-cold system and therapeutic procedures mediate medicinal plant use in san miguel tulancingo, Oaxaca, Mexico. J. Ethnopharmacol..

[128] Pérez-Ortega G., Angeles-López G.E., Argueta-Villamar A., González-Trujano M.E. (2017). Preclinical evidence of the anxiolytic and sedative-like activities of *Tagetes erecta* L. Reinforces its ethnobotanical approach. Biomed. Pharmacother..

[129] Hitziger M., Heinrich M., Edwards P., Pöll E., Lopez M., Krütli P. (2016). Maya phytomedicine in guatemala—Can cooperative research change ethnopharmacological paradigms?. J. Ethnopharmacol..

[130] Alonso-Castro J., Maldonado-Miranda J., Zarate-Martinez A., Jacobo-Salcedo M., Fernández-Galicia C., Alejandro Figueroa-Zuñiga L., Abel Rios-Reyes N., Angel de León-Rubio M., Andrés Medellín-Castillo N., Reyes-Munguia A. (2012). Medicinal plants used in the huasteca potosina, México. J. Ethnopharmacol..

[131] Maity N., Nema N.K., Abedy M.K., Sarkar B.K., Mukherjee P.K. (2011). Exploring *Tagetes erecta* Linn flower for the elastase, hyaluronidase and mmp-1 inhibitory activity. J. Ethnopharmacol..

[132] Ballabh B., Chaurasia O.P., Ahmed Z., Singh S.B. (2008). Traditional medicinal plants of cold desert ladakh-used against kidney and urinary disorders. J. Ethnopharmacol..

[133] Shinde N.V., Kanase K.G., Shilimkar V.C., Undale V.R., Bhosale A.V. (2009). Antinociceptive and anti-inflammatory effects of solvent extracts of *Tagetes erectus* Linn (Asteraceae). Trop. J. Pharm. Res..

[134] Gras A., Garnatje T., Ibanez N., Lopez-Pujol J., Nualart N., Valles J. (2017). Medicinal plant uses and names from the herbarium of Francesc Bolos (1773–1844). J. Ethnopharmacol..

[135] Rasoanaivo P., Petitjean A., Ratsimamanga-Urverg S., Rakoto-Ratsimamanga A. (1992). Medicinal plants used to treat malaria in Madagascar. J. Ethnopharmacol..

[136] Mahomoodally M.F. (2014). A quantitative ethnobotanical study of common herbal remedies used against 13 human ailments categories in mauritius. Afr. J. Tradit. Complement. Altern. Med..

[137] Samoisy A.K., Mahomoodally F. (2016). Ethnopharmacological appraisal of culturally important medicinal plants and polyherbal formulas used against communicable diseases in Rodrigues Island. J. Ethnopharmacol..

[138] Kumar R., Bharati K.A. (2013). New claims in folk veterinary medicines from uttar pradesh, India. J. Ethnopharmacol..

[139] Pande P.C., Tiwari L., Pande H.C. (2007). Ethnoveterinary plants of uttaranchal—A review. Indian J. Tradit. Knowl..

[140] Kidane B., van der Maesen L.J., van Andel T., Asfaw Z. (2014). Ethnoveterinary medicinal plants used by the maale and ari ethnic communities in southern ethiopia. J. Ethnopharmacol..

[141] Santos D.C.D., Schneider L.R., da Silva Barboza A., Diniz Campos Â., Lund R.G. (2017). Systematic review and technological overview of the antimicrobial activity of *Tagetes minuta* and future perspectives. J. Ethnopharmacol..

[142] Wanzala W., Ogoma S.B. (2013). Chemical composition and mosquito repellency of essential oil of *Tagetes minuta* from the southern slopes of mount elgon in western Kenya. J. Essent. Oil Bear. Plants.

[143] Uhlenbroeck J., Bijloo J. (1958). Investigation on nematicides. Isolation and structure of a nematicidal principle occurring in *tagetes* roots. Recuid des Travaux Chimiques des Pays.

[144] Swarup G., Sharma A. (1967). Effect of root extracted of *Asparagus racemosus* and *Tagetes erecta* on hatching of eggs of *Meloidogyne javanica* and *M. arenaria*. Indian J. Exp. Biol..

[145] Munoz L., Roger O., Lopez C., Arias R., Francisco P., Calzad J. (1982). Potential natural nematicides from plant of the genus *tagetes (compositae)*. El Ingeniero en Nanotecnología y Ciencias Químicas.

[146] Fujimoto T., Kyo M., Miyauchi Y., Mayama S. (1990). Nematocidal activity and α-terthiophene content in marigold callus. Plant Tissue Cult. Lett..

[147] Marotti I., Marotti M., Piccaglia R., Nastri A., Grandi S., Dinelli G. (2010). Thiophene occurrence in different *tagetes* species: Agricultural biomasses as sources of biocidal substances. J. Sci. Food Agric..

[148] Sarin R. (2004). Insecticidal activity of callus culture of *Tagetes erecta*. Fitoterapia.

[149] Nikkon F., Habib M., Karim M., Ferdousi Z., Rahman M., Haque M. (2009). Insecticidal activity of flower of *Tagetes erecta* L. against *Tribolium castaneum* (Herbst). Res. J. Agric. Biol. Sci..

[150] Ravikumar P. (2010). Chemical examination and insecticidal properties of *Tagetes erecta* and *Tagetes patula*. Asian J. Biol. Sci..

[151] Salinas-Sanchez D.O., Aldana-Llanos L., Valdes-Estrada M.E., Gutierrez-Ochoa M., Valladares-Cisneros G., Rodriguez-Flores E. (2012). Insecticidal activity of *Tagetes erecta* extracts on *Spodoptera frugiperda* (Lepidoptera: Noctuidae). Fla. Entomol..

[152] Santos P., Santos V., Mecina G., Andrade A., Fegueiredo P., Moraes V., Silva L., Silva R. (2016). Insecticidal activity of *tagetes* sp. On *Sitophilus zeamais* mots. Int. J. Environ. Agric. Res..

[153] Santos P., Santos V., Mecina G., Andrade A., Fegueiredo P., Moraes V., Silva L., Silva R. (2015). Phytotoxicity of *Tagetes erecta* L. and *Tagetes patula* L. On plant germination and growth. S. Afr. J. Bot..

[154] Zoubiri S., Baaliouamer A. (2014). Potentiality of plants as source of insecticide principles. J. Saudi Chem. Soc..

[155] Arora K., Batish D., Kohli R., Singh H. (2017). Allelopathic impact of essential oil of *Tagetes minuta* on common agricultural and wasteland weeds. Innov. J. Agric. Sci..

[156] Coelho L.C., Bastos A.R.R., Pinho P.J., Souza G.A., Carvalho J.G., Coelho V.A.T., Oliveira L.C.A., Domingues R.R., Faquin V. (2017). Marigold (*Tagetes erecta*): The potential value in the phytoremediation of chromium. Pedosphere.

[157] Tereschuk M.L., Riera M.V.Q., Castro G.R., Abdala L.R. (1997). Antimicrobial activity of flavonoids from leaves of *Tagetes minuta*. J. Ethnopharmacol..

[158] Tereschuk M.L., Baigori M.D., Abdala L.R. (2003). Antibacterial activity of *Tagetes terniflora*. Fitoterapia.

[159] Hernandez T., Canales M., Flores C., Garcia A.M., Duran A., Avila J.G. (2008). Antimicrobial activity of *Tagetes lucida*. Pharm. Biol..

[160] Cespedes C.L., Avila J.G., Martinez A., Serrato B., Calderon-Mugica J.C., Salgado-Garciglia R. (2006). Antifungal and antibacterial activities of mexican tarragon (*Tagetes lucida*). J. Agric. Food Chem..

[161] Dunkel F.V., Jaronski S.T., Sedlak C.W., Meiler S.U., Veo K.D. (2010). Effects of steam-distilled shoot extract of *Tagetes minuta* (Asterales: Asteraceae) and entomopathogenic fungi on larval *Tetanops myopaeformis*. Environ. Entomol..

[162] Thembo K.M., Vismer H.F., Nyazema N.Z., Gelderblom W.C., Katerere D.R. (2010). Antifungal activity of four weedy plant extracts against selected mycotoxigenic fungi. J. Appl. Microbiol..

[163] Behidj-Benyounes N., Bennaamane S., Bissaad F., Chebouti N., Mohandkaci H., Abdalaziz N., Iddou S. (2014). Antimicrobial potentials of flavonoids isolated from *Tagetes erecta*. Int. J. Bioeng. Life Sci..

[164] Rhama S., Madhavan S. (2011). Antibacterial activity of the flavonoid, patulitrin isolated from the flowers of *Tagetes erecta* L.. Int. J. PharmTech Res..

[165] Das B., Mishra P. (2011). Antibacterial analysis of crude extracts from the leaves of *Cannabis sativa*. Int. J. Environ. Sci..

[166] Chakraborthy G.S. (2009). Antibacterial and antifungal studies of *Tagetes erectus* leaf extracts. J. Pure Appl. Microbiol..

[167] Jain R., Katare N., Kumar V., Samanta A., Goswami S., Shrotri C. (2012). In vitro antibacterial potential of different extracts of *Tagetes erecta* and *Tagetes patula*. J. Nat. Sci. Res..

[168] Kuddus M., Alam M., Chowdhury S., Rumi F., Sikder A., Rashid M. (2012). Evaluation of membrane stabilizing activity, total phenolic content, brine shrimp lethality bioassay, thrombolytic and antimicrobial activities of *Tagetes patula* L.. J. Pharmacogn. Phytochem..

[169] Faizi S., Siddiqi H., Bano S., Naz A., Lubna, Mazhar K., Nasim S., Riaz T., Kamal S., Ahmad A. (2008). Antibacterial and antifungal activities of different parts of *Tagetes patula*: Preparation of patuletin derivatives. Pharm. Biol..

[170] Capunzo M., Brunetti L., Cavallo P., Boccia G., De Caro F., Ieluzzi M. (2003). Antimicrobial activity of dry extracts of *Tagetes lucida* from Guatemala. J. Prev. Med. Hyg..

[171] Senatore F., Napolitano F., Mohamed M.A.H., Harris P.J.C., Minkeni P.N.S., Henderson J. (2004). Antibacterial activity of *Tagetes minuta* L. (Asteraceae) essential oil with different chemical composition. Flavour Fragr. J..

[172] Wanjala W., Wanzala W. (2016). Chapter 90-*Tagetes* (*Tagetes minuta*) oils. Essent. Oils Food Preserv. Flavor Saf..

[173] Salehi B., Valussi M., Jugran A.K., Martorell M., Ramírez-Alarcón K., Stojanović-Radić Z.Z., Antolak H., Kręgiel D., Mileski K.S., Sharifi-Rad M. (2018). Nepeta species: From farm to food applications and phytotherapy. Trends Food Sci. Technol..

[174] Sharifi-Rad M., Roberts T.H., Matthews K.R., Bezerra C.F., Morais-Braga M.F.B., Coutinho H.D.M., Sharopov F., Salehi B., Yousaf Z., Sharifi-Rad M. (2018). Ethnobotany of the genus *Taraxacum*—Phytochemicals and antimicrobial activity. Phytother. Res..

[175] Marvdashti L.M., Abdolshahi A., Hedayati S., Sharifi-Rad M., Iriti M., Salehi B., Sharifi-Rad J. (2018). Pullulan gum production from low-quality fig syrup using *Aureobasidium pullulans*. Cell. Mol. Biol..

[176] Tereschuk M.L., Baigorí M.D., De Figueroa L.I., Abdala L.R. (2004). Flavonoids from Argentine *Tagetes* (Asteraceae) with antimicrobial activity. Methods Mol. Biol..

[177] Romero C.M., Vivacqua C.G., Abdulhamid M.B., Baigori M.D., Slanis A.C., de Allori M.C.G., Tereschuk M.L. (2016). Biofilm inhibition activity of traditional medicinal plants from northwestern Argentina against native pathogen and environmental microorganisms. Rev. Soc. Bras. Med. Trop..

[178] Verghese J. (1998). Focus on xanthophylls from *Tagetes erecta* L. the giant natural color complex-ii. Indian Spices.

[179] Verghese J. (1998). Focus on xanthophylls from *Tagetes erecta* L. the giant natural complex-i. Indian Spices.

[180] Sivel M., Kracmar S., Fisera M., Klejdus B., Kuban V. (2014). Lutein content in marigold flower (*Tagetes erecta* L.) concentrates used for production of food supplements. Czech J. Food Sci..

[181] EFSA (2008). Scientific opinion on the safety, bioavailability and suitability of lutein for the particular nutritional use by infants and young children. EFSA J..

[182] EFSA (2012). Scientific opinion on the substantiation of health claims related to lutein and maintenance of normal vision (id 1603, 1604, further assessment) pursuant to article 13(1) of regulation (ec) no 1924/2006. EFSA J..

[183] FAO (2016). Lutein esters from *Tagetes erecta*. Chemical and Technical Assessment (CTA).

[184] Wang W., Xu H., Chen H., Tai K., Liu F., Gao Y. (2016). In vitro antioxidant, anti-diabetic and antilipemic potentials of quercetagetin extracted from marigold (*Tagetes erecta* L.) inflorescence residues. J. Food Sci. Technol..

[185] Chkhikvishvili I., Sanikidze T., Gogia N., Enukidze M., Machavariani M., Kipiani N., Vinokur Y., Rodov V. (2016). Constituents of French Marigold (*Tagetes patula* L.) flowers protect jurkat t-cells against oxidative stress. Oxid. Med. Cell. Longev..

[186] WHO (2016). International Statistical Classification of Diseases and Related Health Problems 10th Revision (icd-10).

[187] Juarez-Vazquez M.D., Carranza-Alvarez C., Alonso-Castro A.J., Gonzalez-Alcaraz V.F., Bravo-Acevedo E., Chamarro-Tinajero F.J., Solano E. (2013). Ethnobotany of medicinal plants used in xalpatlahuac, guerrero, mexico. J. Ethnopharmacol..

[188] Sahu P., Masih V., Gupta S., Sen D., Tiwari A. (2014). Ethnomedicinal plants used in the healthcare systems of tribes of dantewada, chhattisgarh India. Am. J. Plant Sci..

[189] Sen S., Chakraborty R., Devanna N. (2011). An ethnobotanical survey of medicinal plants used by ethnic people in west and south district of tripura, India. J. For. Res..

[190] Singh P., Attri B. (2014). Survey on traditional uses of medicinal plants of bageshwar valley (Kumaun Himalaya) of uttarakhand, India. Int. J. Conserv. Sci..

[191] Blanco L., Thiagarajan T. (2017). Ethno-botanical study of medicinal plants used by the yucatec maya in the northern district of belize. Int. J. Herb. Med..

[192] Mollik M., Hossan M., Paul A., Taufiq-Ur-Rahman M., Jahan R., Rahmatullah M. (2010). A comparative analysis of medicinal plants used by folk medicinal healers in three districts of Bangladesh and inquiry as to mode of selection of medicinal plants. Ethnobot. Res. Appl..

[193] Bussmann R., Glenn A. (2010). Plants used for the treatment of gastro-intestinal ailments in northern Peruvian ethnomedicine. Arnaldoa.

[194] Gheno-Heredia Y., Nava-Bernal G., Martínez-Campos Á., Sánchez-Vera E. (2011). Las plantas medicinales de la organización de parteras y médicos indígenas tradicionales de ixhuatlancillo, veracruz, México y su significancia cultural. Polibotánica.

[195] Rosas-Pinon Y., Mejia A., Diaz-Ruiz G., Aguilar M.I., Sanchez-Nieto S., Rivero-Cruz J.F. (2012). Ethnobotanical survey and antibacterial activity of plants used in the altiplane region of Mexico for the treatment of oral cavity infections. J. Ethnopharmacol..

[196] Trillo C., Toledo B., Galetto L., Colantonio S. (2010). Persistence of the use of medicinal plants in rural communities of the western arid Chaco [Córdoba, Argentina]. Open Complement. Med. J..

[197] Igwaran A., Iweriebor B.C., Ofuzim Okoh S., Nwodo U.U., Obi L.C., Okoh A.I. (2017). Chemical constituents, antibacterial and antioxidant properties of the essential oil flower of *Tagetes minuta* grown in cala community eastern cape, South Africa. BMC Complement. Altern. Med..

[198] Lambrecht C., Almeida D., Voigt F., Faccin A., Noremberg R., Schiedeck G., Damé L. (2013). Actividad antibacteriana de los extractos de *Cymbopogon citratus*, *Elionurus* sp. y *Tagetes minuta* contra bacterias que causan mastitis. Rev. Cubana de Plantas Med..

[199] Ayub M.A., Hussain A.I., Hanif M.A., Chatha S.A.S., Kamal G.M., Shahid M., Janneh O. (2017). Variation in phenolic profile, β-carotene and flavonoid contents, biological activities of two *Tagetes* species from pakistani flora. Chem. Biodivers..

[200] Shahzadi I., Shah M.M. (2015). Acylated flavonol glycosides from *Tagetes minuta* with antibacterial activity. Front. Pharmacol..

[201] Pimentel E., Castillo D., Del Solar Q., Maurtua D., Villegas L., Díaz C. (2015). Efecto antibacteriano de extractos etanólicos de plantas utilizadas en la tradiciones culinarias andinas sobre microorganismos de la cavidad bucal. Rev. Estomatol. Hered..

[202] Kazibwe Z., Kim D.H., Chun S., Gopal J. (2017). Ultrasonication assisted ultrafast extraction of *Tagetes erecta* in water: Cannonading antimicrobial, antioxidant components. J. Mol. Liq..

[203] Romagnoli C., Bruni R., Andreotti E., Rai M.K., Vicentini C.B., Mares D. (2005). Chemical characterization and antifungal activity of essential oil of capitula from wild indian *Tagetes patula* L.. Protoplasma.

[204] Sesan T., Enache E., Iacomi B., Oprea M., Oancea F., Iacomi C. (2015). Biological action of plant extracts on a fungal plant biostimulant strain of *Trichoderma viride*. Acta Horti Bot. Bucur..

[205] Alzamora L., Morales L., Armas L., Fernández G. (2001). Medicina tradicional en el perú: Actividad antimicrobiana in vitro de los aceites esenciales extraídos de algunas plantas aromáticas. Anal. Fac. Med..

[206] Dutta B.K., Karmakar S., Naglot A., Aich J.C., Begam M. (2007). Anticandidial activity of some essential oils of a mega biodiversity hotspot in india. Mycoses.

[207] Mares D., Tosi B., Poli F., Andreotti E., Romagnoli C. (2004). Antifungal activity of *Tagetes patula* extracts on some phytopathogenic fungi: Ultrastructural evidence on *Pythium ultimum*. Microbiol. Res..

[208] Negro V., Mancini G., Ruggeri B., Fino D. (2016). Citrus waste as feedstock for bio-based products recovery: Review on limonene case study and energy valorization. Bioresour. Technol..

[209] Vieira A.J., Beserra F.P., Souza M.C., Totti B.M., Rozza A.L. (2018). Limonene: Aroma of innovation in health and disease. Chem. Biol. Interact..

[210] Da Silva A.C., Lopes P.M., de Azevedo M.M., Costa D.C., Alviano C.S., Alviano D.S. (2012). Biological activities of alpha-pinene and beta-pinene enantiomers. Molecules.

[211] Marchese A., Barbieri R., Coppo E., Orhan I.E., Daglia M., Nabavi S.F., Izadi M., Abdollahi M., Nabavi S.M., Ajami M. (2017). Antimicrobial activity of eugenol and essential oils containing eugenol: A mechanistic viewpoint. Crit. Rev. Microbiol..

